# Polygenic risk score improves the accuracy of a clinical risk score for coronary artery disease

**DOI:** 10.1186/s12916-022-02583-y

**Published:** 2022-11-07

**Authors:** Austin King, Lang Wu, Hong-Wen Deng, Hui Shen, Chong Wu

**Affiliations:** 1 Department of Statistics, Florida State University, Tallahassee, FL USA; 2 Cancer Epidemiology Division, Population Sciences in the Pacific Program, University of Hawaii Cancer Center, University of Hawaii at Manoa, Honolulu, HI USA; 3 Center of Bioinformatics and Genomics, Department of Global Biostatistics and Data Science, Tulane University, New Orleans, LA USA; 4 Department of Biostatistics, The University of Texas MD Anderson Cancer Center, Houston, TX USA

**Keywords:** Pooled cohort equations, Integrated polygenic risk score, Genomic risk prediction

## Abstract

**Background:**

The value of polygenic risk scores (PRSs) towards improving guideline-recommended clinical risk models for coronary artery disease (CAD) prediction is controversial. Here we examine whether an integrated polygenic risk score improves the prediction of CAD beyond pooled cohort equations.

**Methods:**

An observation study of 291,305 unrelated White British UK Biobank participants enrolled from 2006 to 2010 was conducted. A case–control sample of 9499 prevalent CAD cases and an equal number of randomly selected controls was used for tuning and integrating of the polygenic risk scores. A separate cohort of 272,307 individuals (with follow-up to 2020) was used to examine the risk prediction performance of pooled cohort equations, integrated polygenic risk score, and PRS-enhanced pooled cohort equation for incident CAD cases. The performance of each model was analyzed by discrimination and risk reclassification using a 7.5% threshold.

**Results:**

In the cohort of 272,307 individuals (mean age, 56.7 years) used to analyze predictive accuracy, there were 7036 incident CAD cases over a 12-year follow-up period. Model discrimination was tested for integrated polygenic risk score, pooled cohort equation, and PRS-enhanced pooled cohort equation with reported C-statistics of 0.640 (95% CI, 0.634–0.646), 0.718 (95% CI, 0.713–0.723), and 0.753 (95% CI, 0.748–0.758), respectively. Risk reclassification for the addition of the integrated polygenic risk score to the pooled cohort equation at a 7.5% risk threshold resulted in a net reclassification improvement of 0.117 (95% CI, 0.102 to 0.129) for cases and − 0.023 (95% CI, − 0.025 to − 0.022) for noncases [overall: 0.093 (95% CI, 0.08 to 0.104)]. For incident CAD cases, this represented 14.2% correctly reclassified to the higher-risk category and 2.6% incorrectly reclassified to the lower-risk category.

**Conclusions:**

Addition of the integrated polygenic risk score for CAD to the pooled cohort questions improves the predictive accuracy for incident CAD and clinical risk classification in the White British from the UK Biobank. These findings suggest that an integrated polygenic risk score may enhance CAD risk prediction and screening in the White British population.

**Supplementary Information:**

The online version contains supplementary material available at 10.1186/s12916-022-02583-y.

## Background

Cardiovascular disease (CVD) is a major cause of death worldwide [1]. Risk estimates for CVD have become particularly important for disease prevention and clinical practice [2–5]. Current guidelines from the American College of Cardiology and American Heart Association suggest lipid-lowering treatments for individuals with greater than a 7.5% 10-year absolute risk of developing CVD based on pooled cohort equations (PCE) [6]. Because of the central role of accurate risk estimates in CVD prevention, improving accuracy beyond those already used in clinical practice like PCE could save lives by better identifying high-risk individuals.

Substantial advancements have been made over the past decades in identifying genetic variants associated with coronary artery disease (CAD) [7–10]. Recent advances in polygenic risk scores (PRSs) have sparked a great interest in enhancing disease risk prediction by using the information on millions of variants across the genome [11–14]. However, population health utility of PRSs in CAD risk prediction is controversial. Several studies have shown that PRSs can improve risk prediction accuracy for incident and prevalent CAD cases compared with individual conventional risk factors [15, 16] and combining risk prediction models (like PCE) with PRS improves the performance in terms of net reclassification improvement [17]. On the other hand, several studies [18, 19] integrating PRSs into PCE to assess possible clinical utility have concluded that the current benefits of incorporating PRSs were minimal (although statistically significant) and were not considered clinically significant to warrant their use over current clinical used prediction models. In this manuscript, we investigate why different studies have reached different and controversial conclusions. Specifically, we analyzed UK Biobank data to test the hypothesis that integrated PRSs leveraging multiple newly developed PRS methods, and several genome-wide association study (GWAS) datasets, can improve risk prediction for CAD over the widely used PCE and thus provide improved clinical utility in European populations [9, 20–25]. Furthermore, in secondary analysis, we extended our integrated method to analyze its predictive performance in non-European populations.

## Methods

### Study populations

**Fig. 1:**
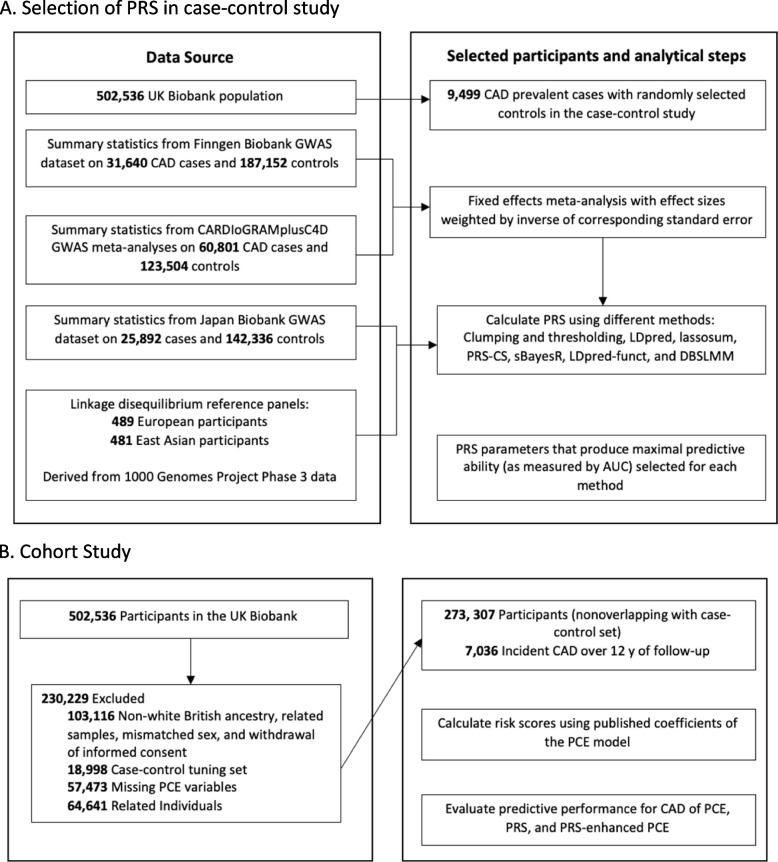
Study design and flowchart for coronary artery disease (CAD). **A** Selection of PRS in the case–control study. **B** The cohort study. To select the parameters for each method with the best discrimination based on the area under the curve (AUC), clumping and thresholding, LDpred, lassosum, PRS-CS, sBayesR, LDpred-funct, and DBSLMM were used to calculate polygenic risk scores (PRSs) on the case–control set consisting of prevalent cases. For these calculations, summary data for three genome-wide association studies (GWAS) on CAD (CARDIoGRAMplusC4D, Finngen Biobank, Japan Biobank) that excluded the UK Biobank and data on linkage disequilibrium were used. The calculated PRSs were applied to a nonoverlapping set of participants from the UK Biobank with no preexisting CAD, aged 40 to 69 at baseline, and who were followed up for incident CAD events. In this population, the pooled cohort equations (PCE) model was calculated and different models (PRS, PCE, PRS-enhanced PCE) were compared in terms of their predictive accuracy based on discrimination, calibration, and reclassification metrics

Our study utilized the UK Biobank which includes 502,536 participants ranging in age from 40 to 69 at baseline recruitment [26]. Biomarker data were collected from stored serum and red blood cells, details of which are described elsewhere [27]. Ethical approval for the UK Biobank study was obtained from the National Health Service’s National Research Ethics Service North West (11/NW/0382). The current research project (application number 48240) was approved by UK Biobank. Our study design is outlined in Fig. 1.

The primary endpoint for our study was CAD, for which several large GWAS results are available [8, 28, 29]. We limited our primary investigation to unrelated White British individuals (as defined by UK Biobank data-field 22,006) to reduce the influence of population heterogeneity and related samples; unrelated individuals were obtained by only keeping individuals with no relative 3rd degree or closer [30]. We further excluded outliers for heterozygosity or genotype missing rates (0.2 > missing rate). Participants with inconsistent reported and genotypic inferred sex and withdrawn consent were likewise removed.

In the secondary analysis, we focused on African and East Asian ancestry participants in the UK Biobank. Following others [31, 32], we used imputed data released by the UK Biobank to determine continental ancestry (African (AFR), East Asian (EAS), European (EUR), South Asian (SAS)) and projected participants onto genetic principal components calculated in the 1000 Genome Project (*N* = 2000: AFR = 504; EAS = 504; EUR = 503; SAS = 489) [33]. We excluded populations identified as African Caribbeans in Barbados (ACB) and Americans of African Ancestry in SW (ASW) from the AFR population and all individuals of American ancestry (AMR) due to their complex admixture patterns. Participants were assigned to ancestries based on likelihoods calculated from their first 5 principal components. Samples were assigned via random forest to an ancestry when their likelihood for a given ancestry was > 0.3. If two ancestries exceeded 0.3, we assigned ancestries as AFR over EUR, SAS over EUR, and EUR over EAS. Participants were excluded if no likelihood was > 0.3 or if 3 ancestry groups were > 0.3 (*n* = 8). The same quality control used in the primary analysis was then applied to the resulting AFR and EAS ancestry populations.

The study population was divided into (1) a case–control study (tuning dataset) established from prevalent CAD cases (see the “Cardiovascular outcome definitions” section for details) and randomly selected controls and (2) an independent prospective cohort study (testing dataset) of participants with no history of CAD at baseline recruitment. The tuning dataset was used for building risk prediction models and the testing dataset was used for unbiasedly evaluating their performance. Of note, there were no overlapping participants between these two datasets, ensuring the testing results were valid.

### Definition of risk score variables

The updated pooled cohort equation (PCE) model, a clinically used risk prediction model, was used as our baseline. We matched variables available in the cohort to the predictors of the updated PCE [3], including information on age, sex, race and ethnicity, smoking status, total and HDL cholesterol, systolic blood pressure, diabetes, and the use of lipid-lowering and blood pressure-lowering medications. Definitions for type 1 and type 2 diabetes, blood pressure-lowering and lipid-lowering medication use, and categorization of smoking status were defined based on UK-recommended QRISK3 scores [34, 35]. Details of variable definitions and protocol for handling missing values are relegated to Additional file 1 [27, 36, 37]. The PCE model categorizes race as a binary variable (“Black” = 1, “White/Other” = 0); therefore, in the secondary analysis, the EAS population was categorized as “Other” for PCE calculations.

### Cardiovascular outcome definitions

The UK Biobank data have been linked to Hospital Episode Statistics (HES) and national death and cancer registries. HES records diagnosis information via International Classification of Diseases (ICD)-9th and 10th Revisions and codes operative procedures via OPCS-4. Death registries include the death date and both primary and secondary causes of death coded in ICD-10. We defined CAD by combining HES, death registries, and operation codes [34, 35], as well as related self-reported diagnoses and previous procedure codes (Additional file 2: Tables S1 and S2). Following others [18], CAD was defined as myocardial infarction, including related sequelae.

The date of the event was determined via recorded episode date, admission date, or operation date indicated in the hospital statistics. For participants with multiple CAD event dates, the earliest recorded date was used as the date of the event. Age of event was determined by self-reported age and calculated age based on the date of the event; when both ages were available, the smaller value was used [15]. Prevalent cases at baseline were defined as individuals with an age of event earlier than the age at UK Biobank enrollment time. Follow-up time was calculated as the number of days from the assessment date until the event of interest (CAD event), a competing cause of death, or censorship date (2020/12/31) occurred.

### Polygenic risk scores (PRSs)

Information on genotyping and imputation has been described in detail elsewhere [27, 38]. Standard quality-control procedures were applied to the imputed UK Biobank genotype data. Briefly, we restricted our analyses to autosomal genetic variants, kept variants with imputation information score (INFO) score > 0.3, minor allele frequency > 1%, Hardy–Weinberg equilibrium *P* > 10^−10^, and genotype missing rate < 10%. We further removed variants with ambiguous strands (A/T or C/G).

PRS for CAD was derived as weighted sums of risk alleles using 3 CAD GWAS datasets (CARDIoGRAMplusC4D, FinnGen Biobank, Japan Biobank) that had no overlap with the present UK Biobank study (Fig. 1) [8, 28, 29]. The 3 GWAS datasets were filtered to only include SNPs present in the imputed UK Biobank data. For all datasets, we aligned β and allele frequencies to the hg19 alternate allele. First, we performed a fixed-effect meta-analysis focused on GWAS datasets with subjects of European ancestry, specifically the CARDIoGRAMplusC4D and FinnGen datasets, using METAL [39]. Second, the PRSs were calculated by using either Japan Biobank data or combined European data and their corresponding population-specific 1000 Genome Project constructed LD reference panels.

Tuning of the PRS was implemented using seven methods: (1) clumping and thresholding using PRSice-2 software (version 2.3.3), (2) LDpred, (3) lassosum, (4) PRS-CS, (5) sBayesR, (6) LDpred-funct, and (7) DBSLMM [20–24, 40, 41]. Detailed information on each PRS method and their associated parameters are described in Additional file 3 [42, 43]. All methods utilized were adjusted for genotype measurement batch and the first five genetic principal components calculated by the UK Biobank. Since different PRS methods and datasets may capture different information, we constructed the integrated PRS by $\sum_{j=1}^{q}{\widehat{\beta }}_{j}{PRS}_{j}$, where ${\widehat{\beta }}_{j}$ is the estimated coefficient of ${PRS}_{j}$ in the logistic regression using the tuning dataset and ${PRS}_{j}$ is the *j*th PRS [44]. Selection of PRS methods for the integrated model was determined based on area under the curve (AUC) results from the tuning dataset. Methods with the largest AUC improvement over the PCE model were selected and analyzed in the testing dataset until the inclusion of additional PRS methods failed to improve the predictive performance of the integrated model. Specifically, we selected the PRS methods with maximal AUC values in the logistic regression model, where CAD status was the outcome and the constructed PRS and baseline variables [PCE, first 5 principal components, and genotype array] were covariates. The AUC values for each PRS method are provided in Tables S3 and S4 in Additional file 4. We assessed the performance of the integrated model against the individual PRS methods in the testing dataset as well as models combining the European meta-analysis data and Japan Biobank data.

### Statistical analysis

Participants were excluded from the study for multiple factors, including missing genetic data, mismatches in reported and genotypic sex, withdrawal of informed consent, and missing predictor values. Using previously published baseline coefficients for each predictor variable and baseline hazard, we calculated the updated pooled cohort equation scores (PCE) [3]. We examined several models as defined in previous studies [18, 19]: (1) PCE, (2) (integrated) PRS for CAD, and (3) PCE and (integrated) PRS. We performed Cox proportional hazard regression using follow-up time as the time variable in the testing data. As a sensitivity analysis, all models were reexamined after removing participants that reported taking lipid-lowering medications at baseline of the UK Biobank study.

We examined the discrimination of each model via Harrell’s C-statistic and its 95% confidence interval [45–47]. In brief, the C-statistic is a measure of the discriminatory power of a risk prediction model, with values ranging from 0.5 (no discrimination) to 1.0 (perfect discrimination). Calibration and recalibration of the baseline models were graphically assessed by comparing observed probabilities via Kaplan–Meier estimates to the mean predicted probability within tenths of the predicted probabilities. During recalibration, the baseline survival function was estimated in the testing cohort and combined with predicted hazard ratios from the validation dataset in a Cox model to obtain recalibrated predicted probabilities [3, 18]. We assessed the recalibration results via the calibration slope and Greenwood-Nam-D’Agostino test [48].

We evaluated risk prediction accuracy using the net reclassification improvement (NRI) [49] at a threshold of 7.5% (clinically used in the USA), continuous NRI, and associated integrated discrimination improvement (IDI) [50]. These metrics quantify how well a new model (PCE plus PRS) reclassifies individuals compared to an old model (PCE); a brief explanation of these metrics can be found in Additional file 3 [51–53].

Statistical analyses were conducted in R software, version 4.0.0 (R Project for Statistical Computing) [54]. Anaconda, version 3.8.3, was also used for PRS methods that utilized Python programming language [55].

## Results

Following the removal of participants with missing data and selecting for only unrelated white British participants, the UK Biobank dataset contained 291,305 participants which were subsequently divided into case–control and cohort study datasets (Fig. 1). The case–control study contained 9499 prevalent CAD cases and an equal number of controls used for tuning of the PRS methods. The independent cohort study was comprised of 272,307 individuals (mean age: 56.7) with 7036 incident cases. Participants with CAD at baseline were not included in the cohort study population. The cohort study had a median follow-up time of 12.33 years (interquartile range, 1.42), while incident CAD cases had a median follow-up time of 5.02 years (interquartile range, 4.07). Baseline characteristics (such as age, smoking status, cholesterol, and systolic blood pressure) were similar for participants included in the cohort analysis and excluded due to missing covariates (Additional file 4: Tables S5-S7).

For the case–control study, each PRS method for CAD was performed across multiple parameter settings to determine optimal values that would be combined for the cohort study. We classified the “optimal” parameter values as those achieving the highest AUC values for that individual method. Specific details on each method’s tuning parameters and individual AUC values were provided in Tables S8 and S9 in Additional file 4 for the European meta-analysis (EUR) and Japan Biobank (Japan) datasets. We combined the PRS for CAD based on the combination of the three GWAS datasets. As expected, because the combined EUR + Japan methods fully utilized all three GWAS datasets and several complementary PRS methods, it achieved the highest AUC [0.641 (95% CI, 0.635–0.648)] and thus we focused on this PRS (denoted by integrated CAD PRS or simply PRS) for the remaining analysis. Our integrated PRS was determined to be weakly, but significantly correlated with CAD events [*r* = 0.0845; *p*-value < 2.2 × 10^−16^]. The maximal integrated CAD PRS model for this study was determined to include the EUR- and Japan-derived clumping and thresholding, LDpred, lassosum, PRS-CS, and LDpred-funct methods. During this step, we evaluated the PRS methods for collinearity concerns and determined the different methods tended to not be highly correlated (Additional file 5: Fig. S1).

**Fig. 2:**
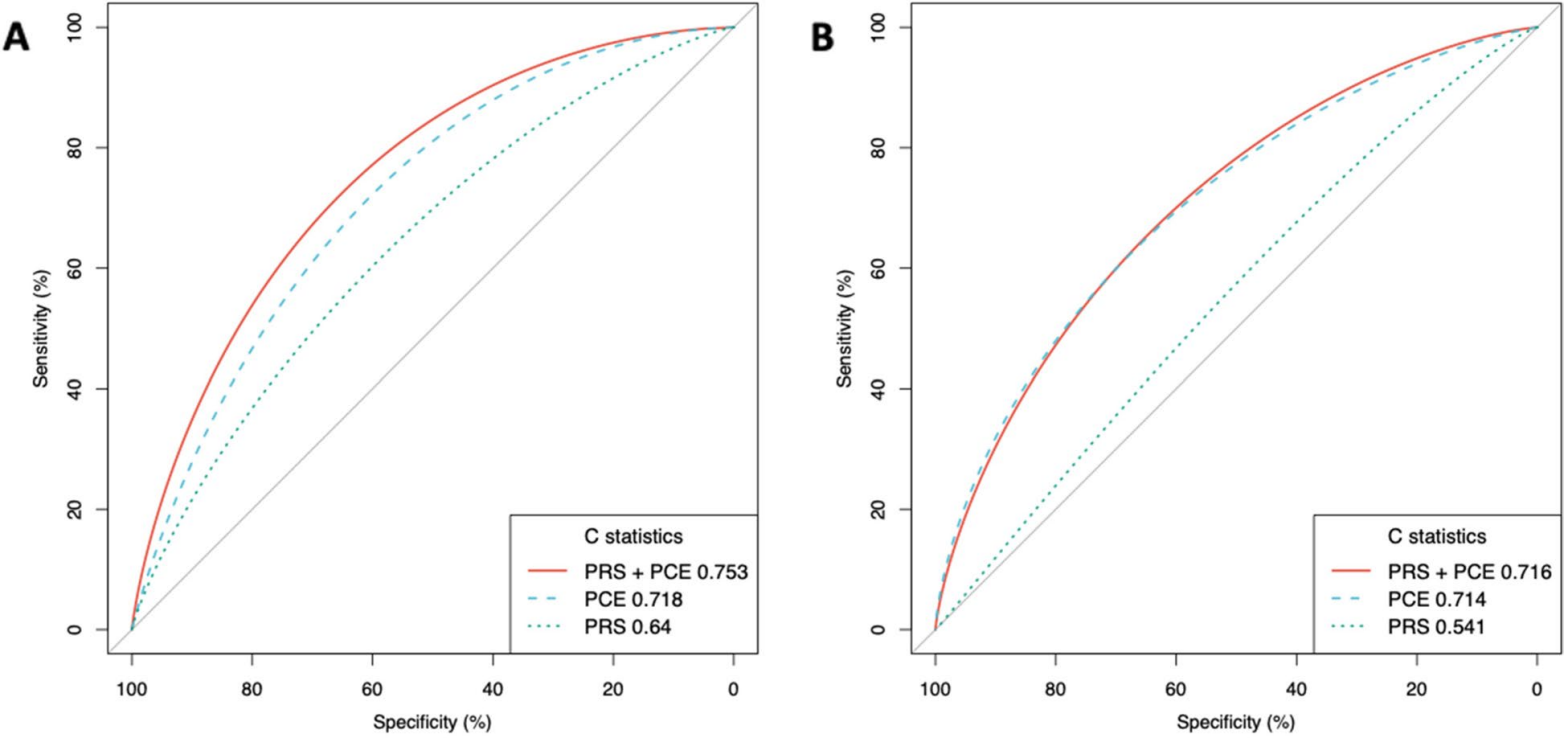
Receiver operator characteristic curves and C-statistics for different models in cohort analyses of White British and African ancestry populations. PCE indicates pooled cohort equation; PRS indicates integrated polygenic risk score. **A** is the White British population of 272,307 individuals over a mean of 12 years of follow-up with 7036 incident CAD cases and **B** is the African ancestry population of 6753 individuals over a mean of 13 years of follow-up with 88 incident CAD cases

In the cohort analysis, following the selection of white British participants, as well as excluding individuals with missing data, and selecting the case–control subjects, 272,307 participants were used. The discrimination of the integrated CAD PRS remained similar as that in the tuning case–control study; the C-statistic for the integrated CAD PRS was 0.640 (95% CI, 0.634–0.646) (Table 1). The discrimination of the PCE (C-statistics, 0.718 [95% CI, 0.713–0.723]) was higher than the integrated CAD PRS. The addition of individual PRSs to the PCE resulted in improved discrimination of the model with PRS-CS applied to the European meta-analysis showing the highest discrimination (C-statistics, 0.749 [95% CI, 0.744–0.754]) (Additional file 4: Tables S10-S12). We observed the most significant improvement in discrimination when the integrated CAD PRS were added to the PCE, showing a C-statistic increase to 0.753 (95% CI, 0.748–0.758), an associated change over the PCE alone of 0.035 (95% CI, 0.03–0.04; *p*-value = 1.91 × 10^−80^) (Table 1 and Fig. 2). We further stratified the population by age group (younger and older than 55 years of age) and sex (men and women) separately and observed higher discrimination in women than men and higher discrimination in the younger age group than in the older age group (Table 1). Participants that were not receiving lipid-lowering medication at baseline were also examined and demonstrated similar discrimination performance (Table 1).

**Table 1:** C-statistics for coronary artery disease for full population and stratified by sex and age group (younger and older than 55 years of age)^A,B^

C-statistic (95% CI)						
	**All**	**Men**	**Women**	**Participants aged < 55 y**	**Participants aged ≥ 55 y**	**Participants not receiving lipid-lowering treatment at baseline**
**A. White British ancestry**						
Participants, no	272,307	124,155	148,152	102,330	169,977	235,172
Cases, no	7036	5093	1943	1276	5760	5091
Polygenic risk score	.64 (.634–.646)	.643 (.636–.651)	.641 (.629–.654)	.69 (.626–.705)	.632 (.625–.639)	.646 (.638–.653)
Pooled cohort equation	.718 (.713–.723)	.663 (.656–.67)	.706 (.695–.717)	.749 (.736–.761)	.665 (.658–.671)	.73 (.724–.737)
Polygenic risk score + pooled cohort equation	.753 (.748–.758)	.714 (.708–.721)	.741 (.73–.751)	.793 (.781–.806)	.705 (.699–.712)	.766 (.76–.772)
**B. African ancestry**						
Participants, no	6753	2901	3852	4528	2225	5896
Cases, no	88	46	42	42	46	63
Polygenic risk score	.542 (.485–.6)	.574 (.494–.654)	.6 (.511–.634)	.548 (.46–.637)	.543 (.462–.624)	.534 (.464–.604)
Pooled cohort equation	.714 (.659–.769)	.674 (.595–.753)	.734 (.653–.815)	.657 (.572–.742)	.721 (.656–.787)	.698 (.628–.768)
Polygenic risk score + pooled cohort equation	.716 (.665–.768)	.695 (.622–.768)	.732 (.654–.81)	.679 (.597–.761)	.696 (.629–.763)	.707 (.64–.774)

^A^Cox proportional hazard models for CAD using recalibrated polygenic risk score, pooled cohort equations, and both combined models

^B^C-statistics shown for combined European meta-analysis + Japan Biobank PRS methods. Results are presented for both White British and African ancestry populations

When evaluating model performance, we compared observed and predicted cumulative incidences of CAD events across each tenth of predicted risk and determined the addition of our integrated PRS method to the baseline model overestimated risk. Following others [17, 18], we recalibrated the model by fitting predicted log-HRs as covariates in the model, resulting in considerable improvement in model calibration (Additional file 5: Fig. S2).

**Fig. 3:**
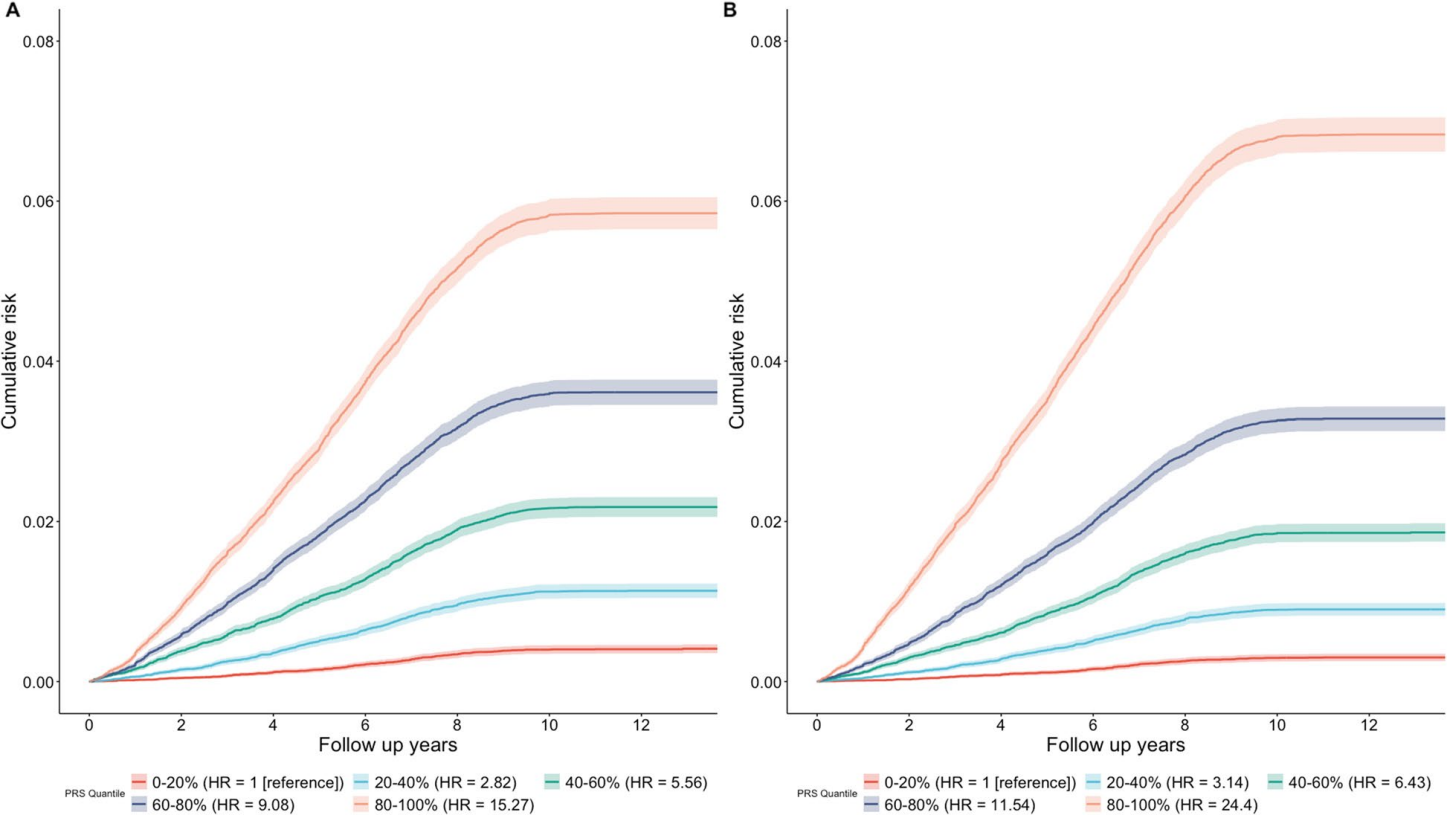
Cumulative absolute risk of developing CAD. Cumulative absolute risk of developing CAD by quintiles of the overall polygenic score in **A** the PCE model and **B** the PRS-enhanced PCE model. The shaded portions correspond to the 95% confidence interval

We investigated the potential of the PRS-enhanced PCE model in the risk assessment of CAD. We found that an individual’s integrated CAD PRS were generally uncorrelated (Pearson correlation coefficient *r*, 0.01) with their PCE, which partially explains why adding integrated CAD PRS to the PCE model (denoted by PRS-enhanced PCE) improves the discrimination power. We evaluated the hazard ratios HR via a Cox regression. The PCE model had an adjusted HR of 1.653 (95% CI: 1.628–1.679) per standard deviation increase (*p* < 0.001) while the PRS-enhanced PCE model reported an adjusted HR of 1.77 (95% CI: 1.745–1.796) per standard deviation increase of PRS (*p* < 0.001). The PRS-enhanced PCE model further improves the discrimination power of the PCE model (Fig. 3). For example, in the PRS-enhanced PCE model, there was a 7.77-fold (95% CI: 7.61- to 7.92-fold) risk of CAD for individuals in the top quintile compared to those in the bottom quintile. The PCE model, in comparison, reported a 5.29-fold (95% CI: 5.21- to 5.39-fold) risk of CAD between the top and bottom quintiles.

**Fig. 4:**
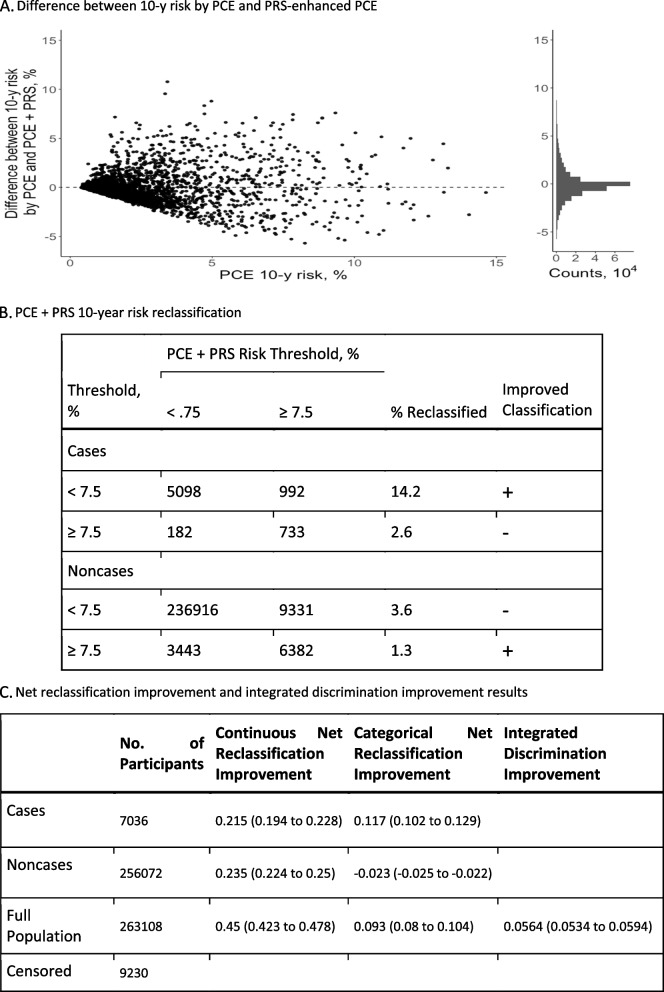
Change in predicted probabilities and risk reclassification. **A** Difference between 10-year risk by PCE and PRS-enhanced PCE. **B** PCE + PRS 10-year risk reclassification. **C** Net reclassification improvement and integrated discrimination improvement results. **A** Change in the predicted probabilities of the recalibrated pooled cohort equations (PCE) model after the addition of polygenic risk scores (PRSs) for CAD. The *x*-axis shows the predicted probability from the baseline PCE model. The *y*-axis is the difference in 10-year risk probabilities of a CAD event between the PRS-enhanced model and the baseline PCE model. The scatterplot has a random draw of 1% of the participants shown. The histogram *x*- and *y*-axes are based on the full population. **B** Reclassification table of predicted probabilities by PCE and PRS-enhanced PCE models at 7.5% threshold. Rows indicating an improved classification with the PRS-enhanced PCE model are marked by a plus sign while rows indicating a deteriorated classification are marked by a minus sign. **C** Table of net reclassification improvement (NRI) and integrated discrimination improvement (IDI). NRI^a^ is defined in the continuous case as the sum of proportions of cases and noncases with improved combined score minus the sum of proportions with a deteriorated combined score. In the categorical case, NRI is defined by change at a 7.5% threshold predicted probability. A positive NRI indicates a better combined score overall. IDI^b^ measures the difference of average probabilities of an event in cases and noncases. A larger IDI indicates more discrimination in the combined score. ^a^NRI = P(up|case) − P(down|case) − P(up|noncase) + P(down|noncase). ^b^IDI = P_PCE+PRS_(case) − P_PCE+PRS_(noncase) − P_PCE_(case) + P_PCE_(noncase)

After adding PRS for CAD to the PCE model, predicted risk changed by greater than 1% for 35.5% of participants while changing by 5% or greater for 1.9% of participants (Fig. 4A). There were 7005 incident CAD cases and 256,072 noncases at the 10-year follow-up; 9230 individuals were censored due to lack of disease or follow-up at 10 years. At the suggested 7.5% risk threshold, 992 of 7005 cases (14.2%) were correctly reclassified to the higher-risk category and 182 of 7005 cases (2.6%) were incorrectly moved to the lower-risk category. For noncase participants, 3443 of 256,072 (1.3%) were correctly moved down to the lower-risk category while 9331 of 256,072 (3.6%) were incorrectly moved up to the high-risk category (Fig. 4B).

When comparing the integrated PRS for CAD model to the PCE model, the NRI for cases was 11.7% (95% CI, 10.2 to 12.9%) and − 2.3% (95% CI, − 2.5 to − 2.2%) for noncases (Fig. 4C). Following the addition of the integrated CAD PRS to PCE, the IDI metric indicated an increase in risk difference between cases and noncases of 0.056 (95% CI, 0.053 to 0.059) (Fig. 4C). Stratification by sex indicated higher NRI improvement in men over women; stratification by age group saw similar overall NRI improvement (Additional file 4: Table S13).

### Secondary analyses

There were 6971 participants in the AFR ancestry population that were divided into case–control and cohort datasets. The case–control dataset consisted of 109 prevalent CAD cases and an equal number of controls. The cohort population was composed of 6753 participants (median follow-up: 12.75, interquartile range: 1.25) in which 88 incident CAD cases were observed (median follow-up: 5.97, interquartile range: 3.3). Baseline characteristics are presented in Tables S14-S16 in Additional file 4.

In the case–control analysis, the optimized integrated CAD PRS model that achieved the highest AUC (0.717 [95% CI, 0.644–0.769]) was determined to include the EUR clumping and thresholding, LDpred, PRS-CS, and LDpred-funct methods as well as the Japan LDpred, PRS-CS, and sBayesR methods. In the cohort analysis, the integrated CAD PRS C-statistic was 0.542 (95% CI, 0.485–0.6) (Fig. 1). Discrimination of the PCE model (0.714 [95% CI, 0.659–0.769]) outperformed the integrated CAD PRS. In contrast to the White British population, the incremental value of the addition of the integrated CAD PRS to the PCE model was minimal (increase in C-statistic, 0.002 [95% CI, 0.006 to − 0.001; *p*-value = 0.824]) (Table 1). We further stratified by gender and age and observed higher discrimination in women and in the older age group; however, we noticed a slightly greater improvement in discrimination with the addition of our integrated CAD PRS in both men and the younger age group. Participants not on lipid-lowering medication at baseline saw slightly higher, but still minimal discrimination improvement than the full population (Table 1). C-statistics for the European meta-analysis and Japan Biobank datasets are presented in Table S17 in Additional file 4. NRI and IDI metrics were likewise minimal and incrementally smaller than in the White British population (Additional file 4: Table S18).

There were 2274 participants in the EAS population that were similarly divided into case–control and cohort datasets. The case–control dataset consisted of 31 prevalent cases and matching number of controls, while the cohort dataset consisted of 2212 individuals (median follow-up: 13.08; interquartile range: 1.5) in which 27 incident CAD cases were observed (median follow-up: 4.85; interquartile range: 3.51). Baseline characteristics for the case–control and cohort datasets, as well as excluded participants, can be found in Tables S19-S21 in Additional file 4.

C-statistics and NRI performance metrics for the EAS population are presented in Tables S22-S24 in Additional file 4. In the case–control study, an optimized CAD PRS model achieved the highest AUC (0.801 [95% CI, 0.726–0.875]) when incorporating the EUR LDpred, LDpredfun, and DBSLMM methods as well as the Japan clumping and thresholding, LDpred, and LDpredfun methods. Discrimination of the PCE model (0.774 [95% CI, 0.706–0.841]) and the PCE model with the addition of the integrated CAD PRS (0.799 [95% CI, 0.726–0.872]) were both higher in the EAS population compared to the White British and AFR populations (Additional file 4: Table S22). However, the incremental value of model performance was determined not to be significant (increase in C-statistic 0.025 [95% CI, 0.02–0.31; *p*-value = 0.209]). Stratification by gender and age group demonstrated the same trend as that in European and African, with higher discrimination observed in women and the under 55 age group. C-statistic results for the European meta-analysis and Japan Biobank datasets are presented in Table S23 in Additional file 4. Participants not receiving lipid-lowering medication had similar discrimination improvements. As the incremental value of model performance was minimal, it was expected that the reported NRI confidence intervals would overlap zero (Additional file 4: Table S24).

## Discussion

In our analysis, the addition of genetic information to the PCE clinical risk score was associated with a moderate improvement in predictive accuracy for CAD. The addition of PRS to the baseline PCE model resulted in a 3.5% improvement in model concordance as well as a 9.3% net reclassification improvement (NRI) of incident CAD cases and noncases over the baseline PCE model at a 7.5% risk threshold. In comparison, the integrated risk tool and Elliott et al. [17, 18] achieved 2.7% (in the European population) and 4.0% (in all UK Biobank subjects) improvement in terms of NRI, respectively. While both studies improve the performance by integrating PRS into PCE, they reached different conclusions regarding its clinical utility, highlighting the importance of building a more powerful and accurate risk prediction model.

Our studies are innovative and are different from existing studies evaluating the clinical utility of adding PRS over existing clinical risk models in the following aspects [18, 19, 56, 57]. While matching our definition of CAD to that of a previous study performed with the UK Biobank [18], we were able to take advantage of more recent incident CAD data. We also utilized a stricter definition for our target population in the UK Biobank data as opposed to the entire UK Biobank data, which contain individuals of diverse ancestry. Recent studies have shown population-specific bias and limited use of specific PRS methods when used on non-European populations [58, 59]. We also used three distinct GWAS datasets to build the PRS and integrated results from several advanced and more recent PRS methods [21–24], improving the discrimination power of our integrated CAD PRS.

We found that integrating PRS to the baseline PCE model resulted in significant continuous and categorical NRI. Categorical NRI for incident cases was 11.7% and − 2.3% for noncases. Our model greatly improved reclassification for cases over previous studies [17–19], but resulted in more misclassification in noncase individuals. This difference in performance for noncases may be due in part to model specifications and cohort selection. In contrast to Moseley et al. [19] in which the 2013 PCE model was used, we utilized the updated 2018 PCE as our baseline. The 2013 model was noted to overestimate risk across all risk groups, prompting the development of the updated PCE model [3]. We also used a younger cohort compared to the two cohorts in Moseley et al. (mean age 56.7 years compared to 62.9 and 61.8, respectively). As noted, we included only White British ancestry in our primary cohort. The inclusion of other ethnicities in the cohort may significantly decrease the discrimination power of the PRS constructed. This is shown in our secondary analysis of African ancestry, where the PRS results based on a European ancestry GWAS dataset vastly underperformed compared to the White British population (C-statistics 0.715 vs 0.752, respectively) (Additional file 4: Tables S10 and S17).

Our results suggest an association between predictive accuracy of PRS and incident CAD events that varies based on age and sex. Men showed significantly higher C-statistic improvement than women (0.051 vs 0.035) in the PRS-enhanced PCE model over the baseline PCE model. This is complemented by an 11.6% overall categorical NRI improvement in men compared to 3.6% in women (Additional file 4: Table S13). Recent studies using PRS in the UK Biobank demonstrated comparable results with higher risks for incident CAD in men than women [15, 57, 60]. The improved performance in men may be attributed to the overrepresentation of male CAD cases in the case–control and cohort studies. The use of sex-specific data may lead to the improved prediction accuracy of PRS.

Our results also suggest a genetic component to early-onset cases of CAD and a possible application of PRS in identifying individuals at heightened risk of these cases, as the predictive accuracy of incident CAD cases was higher in participants < 55 years of age. The observed C-statistic for the integrated PRS-enhanced PCE model was 0.793 compared to 0.705 observed in the ≥ 55 age group. This observation supports two recent studies that found high-risk score predictions in genetic variants strongly associated with early-onset CAD (< 40 years old) as well as improved risk classification of early-onset CAD to higher-risk categories that were not classified as such by PCE [9, 61].

When analyzing both the AFR and EAS populations, we found that the addition of our integrated CAD PRS to the PCE model resulted in more varied results. Model discrimination improvement was minimal in the AFR population (C-statistic increase 0.002 [95% CI, 0.006 to − 0.001; *p*-value = 0.824]) with likewise minimal NRI improvement from the integrating the CAD PRS to PCE (Additional file 4: Table S18). The loss of prediction accuracy in the AFR population when training the PRS with a non-AFR GWAS has been demonstrated before with one study finding 42% lower PRS effect sizes in AFR populations compared to EUR populations [62, 63]. This difference in PRS performance may be attributed to greater, on average, genetic distances between African and European ancestry populations [33, 64]. As African populations are among the most under-represented populations in GWAS studies [62], this result highlights an urgent need to collect more GWAS data in these under-represented populations and develop more powerful cross-ancestry PRS methods to achieve more powerful risk prediction.

C-statistics for both the PCE and integrated PRS and PCE models were highest in the EAS population (0.774 and 0.799, respectively). Discrimination improvement was greater (increase in C-statistic 0.025 [95% CI, 0.02–0.31; *p*-value = 0.209]), but the small number of cases limits the extent to which this result can be generalized to a larger population. This is seen in the NRI results where continuous NRI looks promising, but the small size resulted in large confidence intervals that extended to either side of zero (Additional file 4: Table S24). This result aligns with other studies [17] that have found weak results due in part to the lack of EAS participants in the UK Biobank population. While the discriminations observed are the highest of all populations in this analysis, previous studies have pointed out that the PCE tends to overestimate risk in EAS populations [65], and as such, the performance may be elevated due to this and the small incident case sample size. Previous work has demonstrated PRSs in larger EAS populations have had similar performance [10], and as such, further studies in populations with larger EAS populations may yield more significant results.

As a remark, we constructed and evaluated PRS for each ancestry because the PCE model considered different ancestries and different continental ancestries have different linkage disequilibrium (LD) matrices as well as having different minor allele frequencies (MAF) of highly predictive SNPs between different ancestry groups, highlighting the need of constructing PRS for different ancestries. This study design also allowed us to show that constructed PRS was beneficial for White British and highlight the urgent need to improve the diversity of GWAS datasets to reduce the health disparity among populations.

There are limitations in our study. First, our study was conducted in the UK Biobank and is, therefore, limited by the characteristics of the cohort. The UK Biobank cohort is composed of primarily European ancestries (further restricted to White British ancestry in this study) and limited to an age range of 40 to 69 years, restricting its application to other ancestries and age groups. In addition, participants in the UK Biobank assessment tend to be healthier and more well-off compared to the general UK population, [66] and thus, population-level CAD risk may be underestimated in our study. In the secondary analysis, the limited genetic diversity of the UK Biobank cohort is apparent and resulted in significantly smaller tuning and testing. The extent to which our results can be applied to larger non-European ancestries, in particular African and East Asian ancestries, warrants further investigation. These results also highlight the urgency of developing novel cross-ancestry PRS methods [10, 17, 67–69] and using more diverse cohorts to construct PRSs [17]. In addition, as the case–control and cohort analyses are derived from the same study, more broad generalizability of the results requires further investigation. Second, this study included PRS for low frequency and common genetic variants (MAF ≥ 1%) and did not examine the predictive accuracy of rare variants known to affect CAD risk. Third, the algorithm for the selection of CAD cases utilizes self-report, death, and hospital inpatient data for the definition of prevalent and incident CAD cases. As such, misclassification of cases is possible. Fourth, tuning of each PRS method in the case–control study used prevalent CAD cases, which could introduce survival bias. However, simulation studies have demonstrated a limited effect of survival bias on estimated genetic effects of event risks [70]. Fifth, participants with at least 1 missing predictor value were excluded from the study. Excluded participants were not considerably different demographically from those included and thus the missing data are unlikely to have a significant effect on the reported estimates. Sixth, while adding integrated PRS to the PCE model significantly improved the performance of the PCE model in the White British population, such improvement was minimal in African and East Asian populations, which has raised health disparity concerns and impeded its clinical implementation [71]. These results further highlight the urgent need to develop more powerful cross-ancestry PRS methods and collect larger and more diverse GWAS data. Seventh, the current study was focused on evaluating adding PRS to the PCE model and as such was focused on clinical risk factors. However, incorporating socio-demographic, family history, lifestyle, and other environmental variables may further improve the performance of the risk prediction model. Future research that incorporates these factors may further improve the clinical utility of risk models.

## Conclusions

The addition of the integrated CAD PRS to the PCE resulted in a statistically significant improvement in predictive accuracy for incident CAD, especially in individuals under the age of 55 years old in the White British population. It was also associated with moderate improvement in risk reclassification across all subgroups. However, the benefits of adding integrated CAD PRS to the PCE are minimal for the African population. In summary, the inclusion of genetic information to the pooled cohort equation can help improve clinical risk classification and demonstrates the potential for genetic screening in early life to improve clinical risk prediction in the White British population.

## Supplementary Information

**Additional file 1:** Variable definitions and data protocol.

**Additional file 2:** Variable definition codes. **Table S1.** Definition of Codes for Variables in UK Biobank. **Table S2.** Definition of Codes for Coronary Artery Disease.

**Additional file 3:** Polygenic risk score methodology and reclassification metrics.

**Additional file 4:** Additional tables and detailed results. **Table S3.** AUC results for individual PRS methods trained in European meta-analysis dataset. **Table S4.** AUC results for individual PRS methods trained in Japan Biobank dataset. **Table S5.** Descriptive characteristics of tuning dataset, White British population. **Table S6.** Descriptive characteristics of testing dataset, White British population. **Table S7. **Descriptive characteristics of excluded participants, White British population. **Table S8.** Tuning parameter selection for PRS with CAD in European meta-analysis dataset. **Table S9.** Tuning parameter selection for PRS with CAD in Japan Biobank dataset. **Table S10.** C-statistic results for integrated PRS method stratified by GWAS dataset, White British population. **Table S11.** C-statistic results for individual PRS methods in CAD for testing dataset trained in European meta-analysis, White British population. **Table S12.** C-statistic results for individual PRS methods in CAD for testing dataset trained in Japan Biobank dataset, White British population. **Table S13.** Risk reclassification metrics in White British population stratified by gender and age group. **Table S14.** Descriptive characteristics of tuning dataset, African population. **Table S15.** Descriptive characteristics of testing dataset, African population. **Table S16.** Descriptive characteristics of excluded participants, African population. **Table S17.** C-statistic results for integrated PRS method stratified by GWAS dataset, African population. **Table S18.** NRI and IDI metrics, African Population. **Table S19.** Descriptive characteristics of tuning dataset, East Asian population. **Table S20.** Descriptive characteristics of testing dataset, East Asian population. **Table S21.** Descriptive characteristics of excluded participants, East Asian population. **Table S22.** C-statistic results for integrated PRS method, East Asian population. **Table S23.** C-statistic results for integrated PRS method stratified by GWAS dataset, East Asian population. **Table S24.** NRI and IDI metrics, East Asian population.

**Additional file 5:** Additional figures. **Figure S1.** Correlation matrix of PRS methods in tuning dataset, White British population. **Figure S2.** Calibration and recalibration plots in UK Biobank testing dataset.

## Data Availability

UK Biobank data used in this study were available upon UK Biobank approval (https://www.ukbiobank.ac.uk, application number 48240). The summary statistics of genome-wide association studies (GWAS) of FinnGen Biobank can be obtained from https://www.finngen.fi/en/access_results upon registration, CARDIoGRAMplusC4D GWAS data can be directly downloaded at http://www.cardiogramplusc4d.org/data-downloads/, and Japan Biobank GWAS data can be downloaded at https://humandbs.biosciencedbc.jp/en/hum0014-v22#42diseases. 1000 Genomes phase 3 reference panel can be obtained at https://www.internationalgenome.org/data-portal/data-collection/phase-3. The code can be downloaded from https://github.com/ChongWuLab/PolygenicRiskScore_CAD.

## Abbreviations

AFR: African
AUC: Area under the curve
CAD: Coronary artery disease
CI: Confidence interval
CVD: Cardiovascular disease
EAS: East Asian
EUR: European
GWAS: Genome-wide association study
HES: Hospital Episode Statistics
IDI: Integrated discrimination improvement
NRI: Net reclassification improvement
PCE: Pooled cohort equations
PRS: Polygenic risk score
SAS: South Asian
