# Supplementary material for: Polygenic risk score improves the accuracy of a clinical risk score for coronary artery disease
Source: BMC Med. 2022 Nov 7;20:385. doi: 10.1186/s12916-022-02583-y (PMC9639312; doi:10.1186/s12916-022-02583-y)
Supplement: Supplementary file 1 — Additional file 1: Variable definitions and data protocol. [file 12916_2022_2583_MOESM1_ESM.docx]

**Variables included in PCE**

Smoking status was based on self-reported information on current smoking status (“most or all days” and “occasional” smokers), previous smoking status and non-smoking status (previous and non-smoking are coded as non-smokers in PCE). Smoking status was determined to be missing when participant’s answer to the nurse administered questionnaires was “Prefer not to answer.” Mean systolic blood pressure was calculated from two measurements taken seated using an appropriate cuff and an Omron HEM-7015IT digital BP monitor or a manual reading. In cases where digital BP measurement was not available, the manual measurement was utilized. We defined medication status based on nurse administered questionnaires on blood pressure lowering and lipid lowering medications. Serum data was corrected for laboratory dilution effects and excluded if they fell outside the defined UK Biobank specifications. Total serum cholesterol and HDL cholesterol were obtained via enzymatic assays. We calculated their ratio and corrected total and HDL cholesterol for individuals on lipid-lowering medication by dividing total cholesterol by 0.73 and HDL cholesterol by 1.03.

Information on prevalent disease was obtained from nurse administered questionnaires as well as from relevant International Classification of Disease (ICD) or Office of Population Censuses and Surveys Classification of Surgical Operations and Procedures (OPCS-4) codes. These were obtained from in hospital episode statistics (HES) data with a date of event preceding the date of assessment center attendance. Type 1 and 2 diabetes were defined by self-reported diagnoses or from ICD codes in HES data prior to assessment date. In addition, type 2 diabetes was defined by self-reported medications and HbA1c measurements at baseline ≥ 48 mmol/mol. All variables are defined based on ICD-9, ICD-10, OPCS-4, and relevant UK Biobank field codes (see Additional File 2: Table S1 and S2).
