# Supplementary material for: Polygenic risk score improves the accuracy of a clinical risk score for coronary artery disease
Source: BMC Med. 2022 Nov 7;20:385. doi: 10.1186/s12916-022-02583-y (PMC9639312; doi:10.1186/s12916-022-02583-y)
Supplement: Supplementary file 2 — Additional file 2: Variable definition codes. Table S1. Definition of Codes for Variables in UK Biobank. Table S2. Definition of Codes for Coronary Artery Disease. [file 12916_2022_2583_MOESM2_ESM.docx]

**Table S1.** Definition of Variables in UK Biobank

|  | **HES statistics ICD-10 and ICD-9 codes (with date of event < date of visit)** | **Self-reported Data-field 20002** | **Treatment and medication code Data-Field 20003** | **Other data fields** |
| --- | --- | --- | --- | --- |
| **Type 1 diabetes** | ICD-10 E10, O230; ICD-9 25001, 25011, 25021, 25031, 25041, 25051, 25061, 25071, 25081, 25091, 25003, 25013, 25023, 25033, 25043, 25053, 25063, 25073, 25083, 25093 | 1222 |  |  |
| **Type 2 diabetes** | ICD-10 E11, O231; ICD-9 25000, 25010, 25020, 25030, 25040, 25050, 25060, 25070, 25080, 25090, 25002, 25012, 25022, 25032, 25042, 25052, 25062, 25072, 25082, 25092 | 1223, 1220 | 1140868902, 1140874646, 1140874674, 1140874718, 1140874744, 1140883066, 1140884600, 1141152590, 1141157284, 1141168660, 1141171646, 1141173882, 1141189090 | Biobank field 2443 = 1; HbA1c measurement ≥ 48 mmol/mol |
| **Lipid lowering medication** |  |  | 1140861954, 1140861958, 1140888594, 1140888648, 1141146234, 1141192410, 1141192736 |  |
| **Blood pressure lowering medication** |  |  | 1140860192, 1140860292, 1140860696, 1140860728, 1140860750, 1140860806, 1140860882, 1140860904, 1140861088, 1140861190, 1140861276, 1140866072, 1140866078, 1140866090, 1140866102, 1140866108, 1140866122, 1140866138, 1140866156, 1140866162, 1140866724, 1140866738, 1140868618, 1140872568, 1140874706, 1140874744, 1140875808, 1140879758, 1140879760, 1140879762, 1140879802, 1140879806, 1140879810, 1140879818, 1140879822, 1140879826, 1140879830, 1140879834, 1140879842, 1140879866, 1140884298, 1140888552, 1140888556, 1140888560, 1140888646, 1140909706, 1140910442, 1140910614, 1140916356, 1140923272, 1140923336, 1140923404, 114923712, 1140926778, 1140928226, 1141145660, 1141146126, 1141152998, 1141153026, 1141164276, 1141165470, 1141166006, 1141169516, 1141171336, 1141180592, 1141180772, 1141180778, 1141184722, 1141193282, 1141194794, 1141194810 | Biobank fields 6177, 6153 = 2 |

**Table S2.** Definition of Coronary Artery Disease (CAD)

| **ICD-10** | **ICD-9** | **OPCS-4** | **Non-cancer illness code (Biobank field: 20002)** | **Operation code (Biobank field: 20004)** | **Vascular/heart problems diagnosed by doctor (Biobank field: 6150)** |
| --- | --- | --- | --- | --- | --- |
| I21 | 410 | K40.1-4 | 1075: Heart attack/myocardial infarction | 1070: Coronary angioplasty | 1: Heart attack |
| I22 | 411 | K41.1-4 |  | 1095: Coronary artery bypass graft |  |
| I23 | 412 | K45.1-5 |  |  |  |
| I24.1 |  | K49.1-2 |  |  |  |
| I25.2 |  | K49.8-9 |  |  |  |
|  |  | K50.2 |  |  |  |
|  |  | K75.1-4 |  |  |  |
|  |  | K75.8-9 |  |  |  |
