# Supplementary material for: Polygenic risk score improves the accuracy of a clinical risk score for coronary artery disease
Source: BMC Med. 2022 Nov 7;20:385. doi: 10.1186/s12916-022-02583-y (PMC9639312; doi:10.1186/s12916-022-02583-y)
Supplement: Supplementary file 3 — Additional file 3: Polygenic risk score methodology and reclassification metrics. [file 12916_2022_2583_MOESM3_ESM.docx]

**Calculation of Polygenic Risk Scores (PRS)**

A PRS is calculated as the weighted sum of an individual’s risk alleles. Several factors need to be considered during PRS calculations including (i) the weights used for each SNP, (ii) the number of SNPs included in each score, and (iii) the method to account for correlations between SNPs (linkage disequilibrium [LD]).

We looked at seven methods for PRS calculation that approach these concerns differently:

1. Clumping and Thresholding (P +T) – This method is commonly used and estimates SNP effects from the largest GWAS as the SNP weights. LD is accounted for by a clumping algorithm, which produces a subset of independent SNPs while selecting those weight the strongest association to the desired phenotype. The number of SNPs in the subset included in the PRS is selected by applying a P-value threshold to the original GWAS dataset. Typically, calculations are done with multiple P-value thresholds, and in some cases multiple clumping parameters, to maximize the ability of the PRS to predict the desired target phenotype. Polygenic risk scores were calculated over a range of r^2^ thresholds (0.2, 0.4, 0.6, 0.8) and P values (1, 0.5, 0.05, 5e-4, 5e-6, 5e-8).
2. LDpred – The LDpred algorithm uses a Bayesian approach to generate posterior mean effects for each SNP included in the GWAS by conditioning on a genetic prior of effects and LD information from an external reference panel. 1000 Genome Project Phase 3 LD reference panels for each population were used. Only the HapMap3 subset of SNPs were used in the tuning of this method. The prior includes two parameters that need to be explored: the heritability explained by the genotype, which is estimated from the GWAS and accounts for sampling noise and LD, and the fraction of causal markers (the fraction of genetic markers with non-zero effects). A LD radius is also required, indicating the number of SNPs adjusted for on each side of a given SNP. In this study, the LD radius was set to 267 and the fraction of causal markers were examined at p = (1, 0.1, 0.3, 1e-2, 3e-2, 1e-3, 3e-3). Like P+T, multiple fractions of causal markers are investigated to optimize the predictive accuracy of the PRS. LD is approximated using an external reference panel that is specific to ancestry of the GWAS dataset.
3. Lassosum - Lassosum aims to achieve maximum predictive accuracy in similar fashion to LDpred, by optimizing SNP weights and accounting for LD. Lassosum performs this by using a penalized regression model to carry out shrinkage and the selection of SNPs from the GWAS dataset, while using the same external LD reference panel as LDpred. Two model parameters (s and lambda) are set, with the PRS being calculated over a grid of s and lambda values in order to maximize phenotypic prediction. For CAD, lassosum has been shown to perform similar to or better than LDpred. LD reference panels for each GWAS dataset were defined previously and used as recommended by the author. Reference panels were also set to the hg19 genome.
4. PRS-CS - This method is another method that utilizes a high-dimensional Bayesian framework to infer posterior effect sizes of SNPs using genetic prior effect sizes and an external LD reference panel. In contrast to other methods developed, PRS-CS places a continuous shrinkage (CS) prior on SNP effect sizes. This allows for marker-specific adaptive shrinkage (shrinkage on each genetic marker is adaptive to the strength of the association signal in GWAS) and is more robust to different genetic architectures. PRS-CS is also more accurate in modeling local LD patterns. In simulation, PRS-CS has been shown to outperform LDpred, especially as training sample size increases. For CAD, PRS-CS has demonstrated similar predictive accuracy to LDpred. For our study, the European (EUR) and East Asian (EAS) superpopulation LD reference panels constructed using 1000 Genome Project Phase 3 samples provided by the author were implemented. The European meta-analysis dataset used the EUR LD reference panel, and the Biobank Japan dataset used the EAS reference panel. The polygenic risk scores were calculated using four levels of global shrinkage parameter phi = (1e-6, 1e-4, 1e-2, 1) in order to find the optimal phi value during validation.
5. sBayesR – Another method that infers posterior mean effect sizes from a GWAS dataset and a LD matrix. This method assumes a finite number of normal distributions to account for sparsity with probabilities ranging from 1 to C, the number of components in the mixture model. The recommended parameters for this method are to set C = 4 with parameters γ_c_ = (0,0.01,0.1,1), indicating the scaling factor for the mixture component variance. C was set to 0.95, 0.02, 0.02, and 0.01 in our analysis. This method also requires a shrunk LD matrix, which has been provided by the author. Two different matrices were made available: a 1.1M SNP set constructed by restricting 1,365,446 SNPs from HapMap3 to MAF > 0.01 and a 2.8M SNP set constructed by applying LD-pruning to a larger 8 million variant set from the UK Biobank to MAF > 0.01.
6. LDpred-funct - This method is a modified version of LDpred-funct-inf, which itself is a modification of LDpred-inf. LDpred-funct-inf extends LDpred-inf to incorporate functionally informed priors on causal effect sizes using the baseline-LD model. The 1000 Genome Project Phase 3 derived baseline-LD models (v1.1) containing 75 functional annotations estimated by stratified LD score regression was used for both the European meta-analysis and Japan Biobank datasets. The corresponding EUR and EAS frequency files and HapMap3 subset derived weights were likewise used in the tuning of this method. Each trait is used to determine the per-SNP heritability under the baseline-LD model. LDpred-funct further modifies this method by using cross-validation to regularize posterior mean causal effect sizes. SNPs are ranked by their absolute posterior effect sizes and partitioned into K bins. The relative weights of each bin are determined based on their predictive value in the validation dataset. This non-parametric shrinkage allows LDpred-funct to optimize predictive accuracy regardless of the underlying genetic architecture.
7. DBSLMM – Deterministic Bayesian Sparse Linear Mixed Model (DBSLMM) is a method designed to create a scalable PRS method that is computationally less intensive and thus can be used for larger GWAS datasets efficiently. This method relies on the same effect size distribution assumption made in the BSLMM method. A major difference between these two methods is the use of Markov chain Monte Carlo (MCMC) for posterior sampling in BSLMM, leading it to be computationally slow especially as GWAS size increases. DBSLMM instead utilizes a searching algorithm to subset SNPs with potential large effect sizes. Small effect size SNPs are combined to find total effect size. Both subsets are then combined with a block-diagonal SNP correlation matrix. This leads to DBSLMM having a computationally complexity approximately linear respective to both sample size and the number of SNPs. We tuned this method using the EUR and EAS region reference LD panels in a similar manner as other methods. The recommended block information used in the derivation of the polygenic risk scores was obtained from a previously published study. We tested DBSLMM at three folds of heritability levels: 0.8, 1.0. and 1.2, respectively.

**Reclassification Metrics**

We calculated the net reclassification index (NRI) at the current recommended threshold for treatment in the US (7.5%). The associated category-free NRI and integrated discrimination improvement (IDI) were also calculated. The NRI is calculated for nested models, PCE vs PCE + PRS in our study, using a 2x2 classification table at the defined threshold. “Upward” movement in categories is defined as subjects with a better outcome after reclassification while “downward” movement is defined as subjects with a worse reclassification. The improvement in reclassification is calculated as the sum of the differences in proportions in subjects moving up minus those moving down that had an event/outcome, and the proportion of subjects moving down minus those moving up that did not experience the outcome. NRI has been shown to be sensitive to the number and choices of thresholds, and as such the category-free (continuous) NRI has been proposed. The continuous NRI is calculated as the relative increase in the predictive probabilities of subjects that experience the event and the decrease in those who do not. IDI is defined as a measure that integrates the NRI over all possible cut-offs for the probability of the outcome. The IDI is equivalent to the difference in the discrimination slopes between 2 models.
