# Supplementary material for: Polygenic risk score improves the accuracy of a clinical risk score for coronary artery disease
Source: BMC Med. 2022 Nov 7;20:385. doi: 10.1186/s12916-022-02583-y (PMC9639312; doi:10.1186/s12916-022-02583-y)
Supplement: Supplementary file 4 — Additional file 4: Additional tables and detailed results. Table S3. AUC results for individual PRS methods trained in European meta-analysis dataset. Table S4. AUC results for individual PRS methods trained in Japan Biobank dataset. Table S5. Descriptive characteristics of tuning dataset, White British population. Table S6. Descriptive characteristics of testing dataset, White British population. Table S7. Descriptive characteristics of excluded participants, White British population. Table S8. Tuning parameter selection for PRS with CAD in European meta-analysis dataset. Table S9. Tuning parameter selection for PRS with CAD in Japan Biobank dataset. Table S10. C-statistic results for integrated PRS method stratified by GWAS dataset, White British population. Table S11. C-statistic results for individual PRS methods in CAD for testing dataset trained in European meta-analysis, White British population. Table S12. C-statistic results for individual PRS methods in CAD for testing dataset trained in Japan Biobank dataset, White British population. Table S13. Risk reclassification metrics in White British population stratified by gender and age group. Table S14. Descriptive characteristics of tuning dataset, African population. Table S15. Descriptive characteristics of testing dataset, African population. Table S16. Descriptive characteristics of excluded participants, African population. Table S17. C-statistic results for integrated PRS method stratified by GWAS dataset, African population. Table S18. NRI and IDI metrics, African Population. Table S19. Descriptive characteristics of tuning dataset, East Asian population. Table S20. Descriptive characteristics of testing dataset, East Asian population. Table S21. Descriptive characteristics of excluded participants, East Asian population. Table S22. C-statistic results for integrated PRS method, East Asian population. Table S23. C-statistic results for integrated PRS method stratified by GWAS dataset, East Asian popula [file 12916_2022_2583_MOESM4_ESM.docx]

**Table S3.** Area Under the Curve (AUC) Results for CAD Using European Meta-analysis dataset and Recalibrated Models in the PCE Prospective Cohort, Primary Analysis. Results are presented for full population and stratified by gender and age group (below or above 55 years of age). Results for the sensitivity analysis using only participants with no reported lipid-lowering treatment at baseline also shown.

| A. Full Population (N=272,307; 7036 cases) | | | | | | | |
| --- | --- | --- | --- | --- | --- | --- | --- |
|  | **Clumping and Thresholding** | **LDpred** | **lassosum** | **PRS-CS** | **sBayesR** | **LDpred-funct** | **DBSLMM** |
| **PRS** | 0.604 (0.598-0.611) | 0.632 (0.625-0.638) | 0.629 (0.623-0.636) | 0.631 (0.625-0.638) | 0.604 (0.598-0.611) | 0.622 (0.615-0.628) | 0.524 (0.517-0.531) |
| **PCE** | 0.718 (0.712-0.723) | 0.718 (0.712-0.723) | 0.718 (0.712-0.723) | 0.718 (0.712-0.723) | 0.718 (0.712-0.723) | 0.718 (0.712-0.723) | 0.718 (0.712-0.723) |
| **PRS + PCE** | 0.738 (0.733-0.743) | 0.749 (0.744-0.755) | 0.749 (0.744-0.754) | 0.75 (0.745-0.755) | 0.738 (0.733-0.743) | 0.744 (0.739-0.75) | 0.718 (0.713-0.724) |
| B. Men (N=124,155; 5093 cases) | | | | | | | |
|  | **Clumping and Thresholding** | **LDpred** | **lassosum** | **PRS-CS** | **sBayesR** | **LDpred-funct** | **DBSLMM** |
| **PRS** | 0.608 (0.601-0.616) | 0.634 (0.627-0.642) | 0.633 (0.625-0.64) | 0.635 (0.627-0.643) | 0.607 (0.6-0.615) | 0.623 (0.615-0.631) | 0.515 (0.507-0.523) |
| **PCE** | 0.663 (0.656-0.67) | 0.663 (0.656-0.67) | 0.663 (0.656-0.67) | 0.663 (0.656-0.67) | 0.663 (0.656-0.67) | 0.663 (0.656-0.67) | 0.663 (0.656-0.67) |
| **PRS + PCE** | 0.69 (0.683-0.697) | 0.705 (0.698-0.712) | 0.705 (0.698-0.712) | 0.706 (0.699-0.713) | 0.689 (0.682-0.697) | 0.698 (0.691-0.705) | 0.661 (0.654-0.668) |
| C. Women (N=148,152; 1943 cases) | | | | | | | |
|  | **Clumping and Thresholding** | **LDpred** | **lassosum** | **PRS-CS** | **sBayesR** | **LDpred-funct** | **DBSLMM** |
| **PRS** | 0.602 (0.59-0.615) | 0.632 (0.62-0.645) | 0.623 (0.61-0.635) | 0.63 (0.617-0.643) | 0.602 (0.589-0.614) | 0.623 (0.61-0.635) | 0.527 (0.514-0.54) |
| **PCE** | 0.705 (0.694-0.716) | 0.705 (0.694-0.716) | 0.705 (0.694-0.716) | 0.705 (0.694-0.716) | 0.705 (0.694-0.716) | 0.705 (0.694-0.716) | 0.705 (0.694-0.716) |
| **PRS + PCE** | 0.724 (0.714-0.735) | 0.736 (0.725-0.747) | 0.732 (0.721-0.743) | 0.736 (0.726-0.747) | 0.724 (0.714-0.735) | 0.731 (0.72-0.741) | 0.705 (0.694-0.715) |
| D. Aged < 55 years old (N=102,330; 1276 cases) | | | | | | | |
|  | **Clumping and Thresholding** | **LDpred** | **lassosum** | **PRS-CS** | **sBayesR** | **LDpred-funct** | **DBSLMM** |
| **PRS** | 0.641 (0.626-0.657) | 0.676 (0.661-0.691) | 0.671 (0.656-0.686) | 0.676 (0.661-0.691) | 0.641 (0.626-0.657) | 0.663 (0.648-0.678) | 0.542 (0.525-0.558) |
| **PCE** | 0.749 (0.737-0.762) | 0.749 (0.737-0.762) | 0.749 (0.737-0.762) | 0.749 (0.737-0.762) | 0.749 (0.737-0.762) | 0.749 (0.737-0.762) | 0.749 (0.737-0.762) |
| **PRS + PCE** | 0.771 (0.758-0.783) | 0.784 (0.772-0.796) | 0.784 (0.771-0.796) | 0.785 (0.773-0.798) | 0.771 (0.758-0.783) | 0.78 (0.768-0.793) | 0.747 (0.735-0.76) |
| E. Aged ≥ 55 years old (N=169,977; 5,760 cases) | | | | | | | |
|  | **Clumping and Thresholding** | **LDpred** | **lassosum** | **PRS-CS** | **sBayesR** | **LDpred-funct** | **DBSLMM** |
| **PRS** | 0.6 (0.593-0.607) | 0.625 (.618-0.632) | 0.623 (0.616-0.63) | 0.625 (0.618-0.632) | 0.6 (0.593-0.607) | 0.616 (0.609-0.623) | 0.522 (0.515-0.53) |
| **PCE** | 0.665 (0.658-0.671) | 0.665 (0.658-0.671) | 0.665 (0.658-0.671) | 0.665 (0.658-0.671) | 0.665 (0.658-0.671) | 0.665 (0.658-0.671) | 0.665 (0.658-0.671) |
| **PRS + PCE** | 0.686 (0.68-0.693) | 0.699 (0.693-0.706) | 0.698 (0.692-0.705) | 0.7 (0.693-0.706) | 0.686 (0.68-0.693) | 0.693 (0.686-0.7) | 0.665 (0.658-0.671) |
| F. Participants not receiving lipid-lowering treatment at baseline (N=235,172; 5,091 cases) | | | | | | | |
|  | **Clumping and Thresholding** | **LDpred** | **lassosum** | **PRS-CS** | **sBayesR** | **LDpred-funct** | **DBSLMM** |
| **PRS** | 0.599 (0.591-0.606) | 0.63 (0.623-0.638) | 0.628 (0.62-0.636) | 0.631 (0.623-0.638) | 0.517 (0.509-0.525) | 0.624 (0.617-0.632) | 0.521 (0.513-0.53) |
| **PCE** | 0.73 (0.724-0.737) | 0.73 (0.724-0.737) | 0.73 (0.724-0.737) | 0.73 (0.724-0.737) | 0.73 (0.724-0.737) | 0.73 (0.724-0.737) | 0.73 (0.724-0.737) |
| **PRS + PCE** | 0.746 (0.74-0.752) | 0.758 (0.752-0.764) | 0.758 (0.752-0.764) | 0.759 (0.753-0.765) | 0.73 (0.724-0.736) | 0.756 (0.75-0.762) | 0.729 (0.723-0.736) |

**Table S4.** Area Under the Curve (AUC) Results for CAD Using Japan Biobank dataset and Recalibrated Models in the PCE Prospective Cohort, Primary Analysis. Results are presented for full population and stratified by gender and age group (below or above 55 years of age). Results for the sensitivity analysis using only participants with no reported lipid-lowering treatment at baseline also shown.

| A. Full Population (N=272,307; 7036 cases) | | | | | | | |
| --- | --- | --- | --- | --- | --- | --- | --- |
|  | **Clumping and Thresholding** | **LDpred** | **lassosum** | **PRS-CS** | **sBayesR** | **LDpred-funct** | **DBSLMM** |
| **PRS** | 0.552 (0.545-0.559) | 0.569 (0.562-0.576) | 0.578 (0.571-0.584) | 0.575 (0.569-0.582) | 0.552 (0.546-0.559) | 0.569 (0.563-0.576) | 0.527 (0.52-0.534) |
| **PCE** | 0.718 (0.712-0723) | 0.718 (0.712-0723) | 0.718 (0.712-0723) | 0.718 (0.712-0723) | 0.718 (0.712-0723) | 0.718 (0.712-0723) | 0.718 (0.712-0723) |
| **PRS + PCE** | 0.722 (0.717-0.727) | 0.726 (0.721-0.731) | 0.727 (0.721-0.732) | 0.728 (0.722-0.733) | 0.722 (0.718-0.728) | 0.726 (0.721-0.732) | 0.718 (0.713-0724) |
| B. Men (N=124,155; 5093 cases) | | | | | | | |
|  | **Clumping and Thresholding** | **LDpred** | **lassosum** | **PRS-CS** | **sBayesR** | **LDpred-funct** | **DBSLMM** |
| **PRS** | 0.549 (0.541-0.557) | 0.567 (0.559-0.575) | 0.58 (0.572-0.588) | 0.575 (0.567-0.583) | 0.549 (0.541-0.557) | 0.566 (0.559-0.574) | 0.52 (0.512-0.528) |
| **PCE** | 0.663 (0.656-0.67) | 0.663 (0.656-0.67) | 0.663 (0.656-0.67) | 0.663 (0.656-0.67) | 0.663 (0.656-0.67) | 0.663 (0.656-0.67) | 0.663 (0.656-0.67) |
| **PRS + PCE** | 0.666 (0.659-0.673) | 0.672 (0.665-0.679) | 0.676 (0.669-0.683) | 0.675 (0.668-0.682) | 0.666 (0.66-0.674) | 0.672 (0.665-0.679) | 0.66 (0.653-0.667) |
| C. Women (N=148,152; 1943 cases) | | | | | | | |
|  | **Clumping and Thresholding** | **LDpred** | **lassosum** | **PRS-CS** | **sBayesR** | **LDpred-funct** | **DBSLMM** |
| **PRS** | 0.553 (0.54-0.566) | 0.571 (0.559-0.584) | 0.571 (0.559-0.584) | 0.575 (0.562-0.588) | 0.553 (0.54-0.566) | 0.574 (0.562-0.587) | 0.524 (0.511-0.538) |
| **PCE** | 0.705 (0.694-0.716) | 0.705 (0.694-0.716) | 0.705 (0.694-0.716) | 0.705 (0.694-0.716) | 0.705 (0.694-0.716) | 0.705 (0.694-0.716) | 0.705 (0.694-0.716) |
| **PRS + PCE** | 0.707 (0.696-0.717) | 0.712 (0.701-0.723) | 0.711 (0.701-0.722) | 0.713 (0.702-0.724) | 0.704 (0.693-0.715) | 0.714 (0.703-0.724) | 0.704 (0.693-0.715) |
| D. Aged < 55 years old (N=102,330; 1276 cases) | | | | | | | |
|  | **Clumping and Thresholding** | **LDpred** | **lassosum** | **PRS-CS** | **sBayesR** | **LDpred-funct** | **DBSLMM** |
| **PRS** | 0.587 (0.571-0.602) | 0.605 (0.59-0.621) | 0.619 (0.604-0.634) | 0.617 (0.602-0.633) | 0.587 (0.571-0.602) | 0.607 (0.592-0.622) | 0.55 (0.534-0.566) |
| **PCE** | 0.749 (0.737-0.762) | 0.749 (0.737-0.762) | 0.749 (0.737-0.762) | 0.749 (0.737-0.762) | 0.749 (0.737-0.762) | 0.749 (0.737-0.762) | 0.749 (0.737-0.762) |
| **PRS + PCE** | 0.756 (0.743-0.768) | 0.758 (0.746-0.771) | 0.763 (0.75-0.775) | 0.763 (0.75-0.775) | 0.756 (0.743-0.768) | 0.6 (0.747-0.773) | 0.748 (0.736-0.761) |
| E. Aged ≥ 55 years old (N=169,977; 5,760 cases) | | | | | | | |
|  | **Clumping and Thresholding** | **LDpred** | **lassosum** | **PRS-CS** | **sBayesR** | **LDpred-funct** | **DBSLMM** |
| **PRS** | 0.545 (0.537-0.552) | 0.563 (0.555-0.57) | 0.568 (0.56-0.575) | 0.568 (0.56-0.575) | 0.545 (0.537-0.552) | 0.562 (0.555-0.57) | 0.526 (0.518-0.533) |
| **PCE** | 0.665 (0.658-0.671) | 0.665 (0.658-0.671) | 0.665 (0.658-0.671) | 0.665 (0.658-0.671) | 0.665 (0.658-0.671) | 0.665 (0.658-0.671) | 0.665 (0.658-0.671) |
| **PRS + PCE** | 0.667 (0.661-0.674) | 0.672 (0.665-0.678) | 0.673 (0.666-0.68) | 0.673 (0.666-0.68) | 0.667 (0.661-0.674) | 0.672 (0.665-0.679) | 0.665 (0.658-0.671) |
| F. Participants not receiving lipid-lowering treatment at baseline (N=235,172; 5,091 cases) | | | | | | | |
|  | **Clumping and Thresholding** | **LDpred** | **lassosum** | **PRS-CS** | **sBayesR** | **LDpred-funct** | **DBSLMM** |
| **PRS** | 0.55 (0.542-0.558) | 0.567 (0.559-0.575) | 0.57 (0.562-0.578) | 0.573 (0.565-0.581) | 0.515 (0.507-0.523) | 0.566 (0.558-0.574) | 0.525 (0.517-0.533) |
| **PCE** | 0.73 (0.724-0.737) | 0.73 (0.724-0.737) | 0.73 (0.724-0.737) | 0.73 (0.724-0.737) | 0.73 (0.724-0.737) | 0.73 (0.724-0.737) | 0.73 (0.724-0.737) |
| **PRS + PCE** | 0.733 (0.727-0.74) | 0.738 (0.731-0.744) | 0.739 (0.732-0.745) | 0.739 (0.733-0.745) | 0.729 (0.722-0.735) | 0.738 (0.732-0.744) | 0.73 (0.723-0.736) |

**Table S5.** Descriptive Characteristics of Tuning (Case-Control) Set (N=18,998)

|  | **Men** | **Women** |
| --- | --- | --- |
| **N** | 12043 | 6955 |
| **Age (years) (mean (SD)) *** | 60.23 (7.21) | 58.16 (7.82) |
| **Mean SBP (mmHg) (mean (SD)) *** | 137.92 (18.57) | 137.62 (18.7) |
| **Smoking category (%) *** |  |  |
| **Non-smoker** | 4652 (38.6) | 3857 (55.5) |
| **Ex-smoker** | 5974 (49.6) | 2450 (35.2) |
| **Current smoker** | 1417 (11.8) | 648 (9.3) |
| **Type 1 diabetes (%) *** | 325 (2.7) | 118 (1.7) |
| **Type 2 diabetes (%) *** | 2621 (21.8) | 775 (11.1) |
| **Blood pressure lowering medication (%) *** | 7877 (65.4) | 2503 (36.0) |
| **Cholesterol (mmol/L) (mean (SD)) *** | 4.82 (1.15) | 5.63 (1.18) |
| **HDL cholesterol (mmol/L) (mean (SD)) *** | 1.2 (0.3) | 1.55 (0.38) |
| **Lipid lowering medication (%) *** | 7230 (60.0) | 1886 (27.1) |

* Variables used in PCE. Current smokers vs non-current smokers and diabetes as a binary variable

**Table S6.** Descriptive Characteristics of PCE Prospective Cohort/Testing Set (N=272,307)

|  | **Full Population** | | **Incident CAD** | |
| --- | --- | --- | --- | --- |
|  | **Men** | **Women** | **Men** | **Women** |
| **N** | 124118 | 148189 | 5093 | 1943 |
| **Age (years) (Mean (SD))** | 56.8 (8.09) | 56.6 (7.88) | 60.44 (6.6) | 61.1 (6.46) |
| **Mean SBP (mmHg) (mean (SD))** | 137.86 (18.66) | 137.84 (18.68) | 138.32 (18.74) | 138.13 (18.45) |
| **Smoking category (%)** |  |  |  |  |
| **Current** | 14580 (11.7) | 12747 (8.6) | 806 (15.8) | 325 (16.7) |
| **Non-current** | 109538 (88.3) | 135442 (91.4) | 4287 (84.2) | 1618 (83.3) |
| **Type 1 Diabetes (%)** | 1260 (1.0) | 1016 (0.7) | 150 (2.9) | 59 (3.0) |
| **Type 2 Diabetes (%)** | 12832 (10.3) | 8962 (6.0) | 1082 (21.2) | 345 (17.8) |
| **Blood pressure lowering medication (%)** | 21506 (17.3) | 15629 (10.5) | 2220 (43.6) | 814 (41.9) |
| **Cholesterol (mmol/L) (mean (SD))** | 5.57 (1.1) | 5.91 (1.12) | 5.28 (1.58) | 5.71 (1.62) |
| **HDL cholesterol (mmol/L) (mean (SD))** | 1.29 (0.31) | 1.6 (0.38) | 1.23 (0.29) | 1.49 (0.36) |
| **Person-years of observation (mean (SD))** | 11.79 (2.27) | 12.13 (1.65) | 4.82 (2.51) | 5.15 (2.5) |
| **Lipid lowering medication (%)** | 21562 (17.4) | 15611 (10.5) | 1466 (28.8) | 479 (24.7) |

**Table S7.** Descriptive Characteristics of the PCE Prospective Cohort and Excluded Participants

|  | **Prospective cohort (N = 272307)** | | **Excluded (N = 43474) *** | | |
| --- | --- | --- | --- | --- | --- |
|  | Men | Women | Men | Women | Missing counts |
| **N** | 124118 | 148189 | 17790 | 20084 | 5600 |
| **Age (years) (Mean (SD))** | 56.8 (8.09) | 56.6 (7.88) | 57.03 (8.08) | 56.65 (7.89) |  |
| **Mean SBP (mmHg) (mean (SD))** | 137.86 (18.66) | 137.84 (18.68) | 137.84 (18.72) | 137.85 (7.89) | 633 |
| **Smoking category (%)** |  |  |  |  | 135 |
| **Current** | 14580 (11.7) | 12747 (8.6) | 2088 (11.8) | 1718 (8.5) |  |
| **Non-current** | 109538 (88.3) | 135442 (91.4) | 15621 (88.2) | 18366 (91.5) |  |
| **Type 1 Diabetes (%)** | 1260 (1.0) | 1016 (0.7) | 211 (1.2) | 161 (0.8) |  |
| **Type 2 Diabetes (%)** | 12832 (10.3) | 8962 (6.0) | 2026 (11.4) | 1257 (6.3) |  |
| **Blood pressure lowering medication (%)** | 21506 (17.3) | 15629 (10.5) | 5159 (29.1) | 4077 (20.3) |  |
| **Cholesterol (mmol/L) (mean (SD))** | 5.57 (1.1) | 5.91 (1.12) | 5.5 (1.13) | 5.89 (1.13) | 15553 |
| **HDL cholesterol (mmol/L) (mean (SD))** | 1.29 (0.31) | 1.6 (0.38) | 1.28 (0.31) | 1.6 (0.38) | 42489 |
| **Person-years of observation (mean (SD))** | 11.79 (2.27) | 12.13 (1.65) | 10.39 (6.23) | 11.39 (4.53) |  |
| **Lipid lowering medication (%)** | 21562 (17.4) | 15611 (10.5) | 3776 (21.3) | 2285 (11.4) |  |

* 5600 participants excluded from calculations due to missing variables on gender

**Table S8.** Association of Different Polygenic Risk Scores (PRS) With Coronary Artery Disease (CAD) in Tuning Case-Control Study and in Prospective Cohort Study in European GWAS meta-analysis

|  |  | **AUC (95% CI) PRS** | |
| --- | --- | --- | --- |
| **Methods** | **P-value/Tuning Parameters** | **Case-control study (N = 9,499)** | **Cohort Study (N = 7,036)** |
| Clumping and Thresholding | R2=0.2, P=0.00000005 | 0.6179 (0.6137-0.6221) | 0.595 (0.5917-0.5982) |
| Clumping and Thresholding | R2=0.2, P=0.000005 | 0.6225 (0.6183-0.6267) | 0.5955 (0.5922-0.5987) |
| Clumping and Thresholding | R2=0.2, P=0.0005 | 0.6230 (0.6188-0.6272) | 0.5976 (0.5944-0.6009) |
| Clumping and Thresholding | R2=0.2, P=0.05 | 0.6005 (0.5963-0.6047) | 0.5835 (0.5802-0.5868) |
| Clumping and Thresholding | R2=0.2, P=0.5 | 0.5966 (0.5924-0.6047) | 0.578 (0.5747-0.5813) |
| Clumping and Thresholding | R2=0.2, P=1 | 0.5962 (0.592-0.6004) | 0.5777 (0.5744-0.5809) |
| Clumping and Thresholding | R2=0.4, P=0.00000005 | 0.6155 (0.6113-0.6197) | 0.593 (0.5897-0.5963) |
| Clumping and Thresholding | R2=0.4, P=0.000005 | 0.6221 (0.6179-.06263) | 0.596 (0.5927-0.5993) |
| Clumping and Thresholding | R2=0.4, P=0.0005 | 0.6291 (0.6249-0.6333) | 0.6039 (0.6007-0.6072) |
| Clumping and Thresholding | R2=0.4, P=0.05 | 0.6146 (0.6104-0.6188) | 0.5941 (0.5908-0.5974) |
| Clumping and Thresholding | R2=0.4. P=0.5 | 0.61 (0.6058-0.6142) | 0.5871 (0.5838-0.5903) |
| Clumping and Thresholding | R2=0.4, P=1 | 0.6094 (0.6052-0.6136) | 0.5865 (0.5832-0.5897) |
| Clumping and Thresholding | R2=0.6, P=0.00000005 | 0.6138 (0.6096-0.6180) | 0.5911 (0.5878-0.5944) |
| Clumping and Thresholding | R2=0.6, P=0.000005 | 0.6236 (0.6194-0.6278) | 0.5982 (0.5949-0.6014) |
| Clumping and Thresholding | R2=0.6, P=0.0005 | 0.6314 (0.6272-0.6356) | 0.6043 (0.6010-0.6075) |
| Clumping and Thresholding | R2=0.6, P=0.05 | 0.6230 (0.6188-0.6272) | 0.5997 (0.5964-0.603) |
| Clumping and Thresholding | R2=0.6, P=0.5 | 0.6169 (0.6127-0.6211) | 0.5924 (0.5892-0.5957) |
| Clumping and Thresholding | R2=0.6, P=1 | 0.6159 (0.6117-0.6201) | 0.5918 (0.5885-0.5951) |
| Clumping and Thresholding | R2=0.8, P=0.00000005 | 0.6069 (0.6027-0.6111) | 0.5852 (0.5819-0.5983) |
| Clumping and Thresholding | R2=0.8, P=0.000005 | 0.6188 (0.6146-0.6230) | 0.5951 (0.5918-0.5983) |
| Clumping and Thresholding | R2=0.8, P=0.0005 | 0.6309 (0.6267-0.6335) | 0.6049 (0.6016-0.6082) |
| Clumping and Thresholding | R2=0.8, P=0.05 | 0.6293 (0.6251-0.6335) | 0.6056 (0.6023-0.6088) |
| Clumping and Thresholding | R2=0.8, P=0.5 | 0.6226 (0.6184-0.6268) | 0.5977 (0.5944-0.6009) |
| Clumping and Thresholding | R2=0.8, P=1 | 0.6217 (0.6175-0.6259) | 0.5972 (0.5939-0.6004) |
| LDpred | p = 0.001 | 0.5322 (0.4874-0.5769) | 0.5198 (0.4806-0.559) |
| LDpred | p = 0.01 | 0.5668 (0.5221-0.6115) | 0.5225 (0.5133-0.5917) |
| LDpred | p = 0.1 | 0.6496 (0.6048-0.6943) | 0.6238 (0.5846-0.663) |
| **Methods** | **P-value/Tuning Parameters** | **Case-control study (N = 9,499)** | **Cohort Study (N = 7,036)** |
| LDpred | p = 1 | 0.6356 (0.5909-0.686) | 0.6123 (0.5731-0.6515) |
| LDpred | p = 0.003 | 0.5323 (0.4876-0.577) | 0.5222 (0.483-0.5614) |
| LDpred | p = 0.03 | 0.6585 (0.6138-0.7033) | 0.6316 (0.5924-0.6708) |
| LDpred | p = 0.3 | 0.6413 (0.5966-0.686) | 0.6168 (0.5776-0.6560) |
| lassosum | s=0.2, lambda=0.001 | 0.5826 (0.5737-0.5916) | 0.5681 (0.5602-0.5761) |
| lassosum | s=0.2, lambda=0.0013 | 0.5885 (0.5795-0.5974) | 0.5732 (0.5652-0.5812) |
| lassosum | s=0.2, lambda=0.0016 | 0.5962 (0.5873-0.6052) | 0.5818 (0.5738-0.5898) |
| lassosum | s=0.2, lambda=0.0021 | 0.6065 (0.5976-0.6155) | 0.5905 (0.5825-0.5985) |
| lassosum | s=0.2, lambda=0.0026 | 0.6201 (0.6111-0.629) | 0.5997 (0.5917-0.6077) |
| lassosum | s=0.2, lambda=0.0034 | 0.6346 (0.6257-0.6436) | 0.6133 (0.6053-0.6212) |
| lassosum | s=0.2, lambda=0.0043 | 0.6487 (0.6397-0.6576) | 0.6225 (0.6146-0.6305) |
| lassosum | s=0.2, lambda=0.0055 | 0.6458 (0.6369-0.6548) | 0.6176 (0.6097-0.6256) |
| lassosum | s=0.2, lambda=0.007 | 0.6281 (0.6191-0.637) | 0.6043 (0.5963-0.6122) |
| lassosum | s=0.2, lambda=0.0089 | 0.6106 (0.6017-0.6196) | 0.5903 (0.5824-0.5983) |
| lassosum | s=0.2, lambda=0.0113 | 0.5955 (0.5865-0.6045) | 0.5778 (0.5698-0.5857) |
| lassosum | s=0.2, lambda=0.0114 | 0.5819 (0.573-0.5909) | 0.5648 (0.5568-0.5728) |
| lassosum | s=0.2, lambda=0.0183 | 0.5714 (0.5624-0.5804) | 0.5545 (0.5465-0.5624) |
| lassosum | s=0.2, lambda=0.0234 | 0.5655 (0.5566-0.5745) | 0.5486 (0.5406-0.5565) |
| lassosum | s=0.2, lambda=0.0298 | 0.5577 (0.5488-0.5667) | 0.5411 (0.5332-0.5491) |
| lassosum | s=0.2, lambda=0.0379 | 0.5569 (0.548-0.5659) | 0.541 (0.533-0.549) |
| lassosum | s=0.2, lambda=0.0483 | 0.5311 (0.5221-0.54) | 0.5191 (0.5111-0.527) |
| lassosum | s=0.2, lambda=0.0616 | 0.5311 (0.5221-0.54) | 0.5191 (0.5111-0.527) |
| lassosum | s=0.2, lambda=0.0785 | 0.5311 (0.5221-0.54) | 0.5191 (0.5111-0.527) |
| lassosum | s=0.2, lambda=0.1 | 0.5311 (0.5221-0.54) | 0.5191 (0.5111-0.527) |
| lassosum | s=0.5, lambda=0.001 | 0.6016 (0.5927-0.6106) | 0.5842 (0.5762-0.5921) |
| lassosum | s=0.5, lambda=0.0013 | 0.6069 (0.598-0.6159) | 0.589 (0.581-0.5969) |
| lassosum | s=0.5, lambda=0.0016 | 0.6131 (0.6041-0.6221) | 0.5951 (0.5871-0.6031) |
| lassosum | s=0.5, lambda=0.0021 | 0.6214 (0.6125-0.6304) | 0.6019 (0.594-0.6099) |
| **Methods** | **P-value/Tuning Parameters** | **Case-control study (N = 9,499)** | **Cohort Study (N = 7,036)** |
| lassosum | s=0.5, lambda=0.0026 | 0.6328 (0.6239-0.6418) | 0.6106 (0.6026-0.6185) |
| lassosum | s=0.5, lambda=0.0034 | 0.6452 (0.6362-0.6541) | 0.6216 (0.6136-0.6296) |
| lassosum | s=0.5, lambda=0.0043 | 0.6538 (0.6449-0.6628) | 0.626 (0.6181-0.634) |
| lassosum | s=0.5, lambda=0.0055 | 0.6454 (0.6364-0.6543) | 0.6178 (0.6099-0.6258) |
| lassosum | s=0.5, lambda=0.007 | 0.6273 (0.6183-0.6363) | 0.6031 (0.5952-0.6111) |
| lassosum | s=0.5, lambda=0.0089 | 0.6103 (0.6013-0.6193) | 0.5895 (0.5815-0.5974) |
| lassosum | s=0.5, lambda=0.0113 | 0.5946 (0.5856-0.6036) | 0.5767 (0.5688-0.5847) |
| lassosum | s=0.5, lambda=0.0114 | 0.5803 (0.5714-0.5893) | 0.5633 (0.5554-0.5713) |
| lassosum | s=0.5, lambda=0.0183 | 0.5702 (0.5613-0.5792) | 0.5535 (0.5456-0.5615) |
| lassosum | s=0.5, lambda=0.0234 | 0.5645 (0.5556-0.5735) | 0.5476 (0.5397-0.5556) |
| lassosum | s=0.5, lambda=0.0298 | 0.5578 (0.5488-0.5667) | 0.5411 (0.5332-0.5491) |
| lassosum | s=0.5, lambda=0.0379 | 0.5576 (0.5486-0.5665) | 0.5411 (0.5331-0.5491) |
| lassosum | s=0.5, lambda=0.0483 | 0.5311 (0.5221-0.54) | 0.5191 (0.5111-0.527) |
| lassosum | s=0.5, lambda=0.0616 | 0.5311 (0.5221-0.54) | 0.5191 (0.5111-0.527) |
| lassosum | s=0.5, lambda=0.0785 | 0.5311 (0.5221-0.54) | 0.5191 (0.5111-0.527) |
| lassosum | s=0.5, lambda=0.1 | 0.5311 (0.5221-0.54) | 0.5191 (0.5111-0.527) |
| lassosum | s=0.9, lambda=0.001 | 0.6279 (0.619-0.6369) | 0.6056 (0.5977-0.6136) |
| lassosum | s=0.9, lambda=0.0013 | 0.6322 (0.6232-0.6411) | 0.6094 (0.6014-0.6173) |
| lassosum | s=0.9, lambda=0.0016 | 0.6375 (0.6285-0.6464) | 0.6139 (0.606-0.6219) |
| lassosum | s=0.9, lambda=0.0021 | 0.6438 (0.6349-0.6528) | 0.6193 (0.6113-0.6272) |
| lassosum | s=0.9, lambda=0.0026 | 0.6513 (0.6423-0.6602) | 0.6253 (0.6174-0.6333) |
| lassosum | s=0.9, lambda=0.0034 | 0.6563 (0.6474-0.6653) | 0.6293 (0.6214-0.6373) |
| lassosum | s=0.9, lambda=0.0043 | 0.6516 (0.6427-0.6606) | 0.6241 (0.6162-0.6321) |
| lassosum | s=0.9, lambda=0.0055 | 0.6366 (0.6276-0.6455) | 0.611 (0.603-0.6189) |
| lassosum | s=0.9, lambda=0.007 | 0.6193 (0.6103-0.6282) | 0.5958 (0.5879-0.6038) |
| lassosum | s=0.9, lambda=0.0089 | 0.603 (0.5941-0.612) | 0.5823 (0.5743-0.5903) |
| lassosum | s=0.9, lambda=0.0113 | 0.5884 (0.5794-0.5973) | 0.5699 (0.5619-0.5779) |
| lassosum | s=0.9, lambda=0.0114 | 0.576 (0.5671-0.585) | 0.5579 (0.5499-0.5659) |
| **Methods** | **P-value/Tuning Parameters** | **Case-control study (N = 9,499)** | **Cohort Study (N = 7,036)** |
| lassosum | s=0.9, lambda=0.0183 | 0.5662 (0.5573-0.5752) | 0.5494 (0.5415-0.5574) |
| lassosum | s=0.9, lambda=0.0234 | 0.5619 (0.553-0.5709) | 0.5448 (0.5368-0.5527) |
| lassosum | s=0.9, lambda=0.0298 | 0.558 (0.549-0.5669) | 0.5412 (0.5332-0.5491) |
| lassosum | s=0.9, lambda=0.0379 | 0.5578 (0.5488-0.5667) | 0.5411 (0.5332-0.5491) |
| lassosum | s=0.9, lambda=0.0483 | 0.5311 (0.5221-0.54) | 0.5191 (0.5111-0.527) |
| lassosum | s=0.9, lambda=0.0616 | 0.5311 (0.5221-0.54) | 0.5191 (0.5111-0.527) |
| lassosum | s=0.9, lambda=0.0785 | 0.5311 (0.5221-0.54) | 0.5191 (0.5111-0.527) |
| lassosum | s=0.9, lambda=0.1 | 0.5311 (0.5221-0.54) | 0.5191 (0.5111-0.527) |
| lassosum | s=1, lambda=0.001 | 0.6221 (0.6132-0.6311) | 0.5982 (0.5902-0.6061) |
| lassosum | s=1, lambda=0.0013 | 0.6231 (0.6141-0.632) | 0.5991 (0.5911-0.607) |
| lassosum | s=1, lambda=0.0016 | 0.6239 (0.6149-0.6329) | 0.5999 (0.5919-0.6078) |
| lassosum | s=1, lambda=0.0021 | 0.6246 (0.6156-0.6335) | 0.6002 (0.5922-0.6082) |
| lassosum | s=1, lambda=0.0026 | 0.6239 (0.6149-0.6328) | 0.5996 (0.5917-0.6076) |
| lassosum | s=1, lambda=0.0034 | 0.6186 (0.6096-0.6276) | 0.5949 (0.587-0.6029) |
| lassosum | s=1, lambda=0.0043 | 0.6086 (0.5996-0.6176) | 0.5864 (0.5785-0.5944) |
| lassosum | s=1, lambda=0.0055 | 0.5972 (0.5882-0.6061) | 0.5776 (0.5697-0.5856) |
| lassosum | s=1, lambda=0.007 | 0.5869 (0.5779-0.5958) | 0.5691 (0.5612-0.5771) |
| lassosum | s=1, lambda=0.0089 | 0.577 (0.5681-0.586) | 0.5598 (0.5519-0.5678) |
| lassosum | s=1, lambda=0.0113 | 0.571 (0.5621-0.58) | 0.5529 (0.545-0.5609) |
| lassosum | s=1, lambda=0.0114 | 0.5662 (0.5572-0.5752) | 0.5471 (0.5392-0.5551) |
| lassosum | s=1, lambda=0.0183 | 0.5606 (0.5516-0.5696) | 0.5427 (0.5348-0.5507) |
| lassosum | s=1, lambda=0.0234 | 0.5593 (0.5503-0.5683) | 0.5418 (0.5339-0.5498) |
| lassosum | s=1, lambda=0.0298 | 0.5584 (0.5494-0.5674) | 0.5412 (0.5332-0.5491) |
| lassosum | s=1, lambda=0.0379 | 0.5578 (0.5488-0.5667) | 0.5411 (0.5332-0.5491) |
| lassosum | s=1, lambda=0.0483 | 0.5311 (0.5221-0.54) | 0.5191 (0.5111-0.527) |
| lassosum | s=1, lambda=0.0616 | 0.5311 (0.5221-0.54) | 0.5191 (0.5111-0.527) |
| lassosum | s=1, lambda=0.0785 | 0.5311 (0.5221-0.54) | 0.5191 (0.5111-0.527) |
| lassosum | s=1, lambda=0.1 | 0.5311 (0.5221-0.54) | 0.5191 (0.5111-0.527) |
| **Methods** | **P-value/Tuning Parameters** | **Case-control study (N = 9,499)** | **Cohort Study (N = 7,036)** |
| PRS-CS | phi = 0.000001 | 0.6379 (0.6071-0.6688) | 0.6120 (0.5877-0.6363) |
| PRS-CS | phi =0.0001 | 0.6621 (0.6312-0.6929) | 0.6315 (0.6072-0.6558) |
| PRS-CS | phi = 0.01 | 0.6393 (0.6084-0.6701) | 0.6147 (0.5904-0.6390) |
| PRS-CS | phi = 1 | 0.6146 (0.5837-0.6454) | 0.5942 (0.5698-0.6185) |
| sBayesR | LD matrix = HapMap3 and 2.8M | 0.5359 (0.5297-0.5421) | 0.5227(0.5053-0.5400) |
| sBayesR | LD matrix = 2.8M variants | 0.5349 (0.5287-0.5411) | 0.5199 (0.5026-0.5373) |
| LDpred-funct | Baseline-LD model with 75 annotations | 0.6460 (0.64-0.652) | 0.6215 (0.6155 - 0.6275) |
| DBSLMM | h2f = 0.8 | 0.5326 (0.5266-0.5386) | 0.5236 (0.5176-0.5296) |
| DBSLMM | h2f = 1 | 0.5326 (0.5266-0.5386) | 0.5236 (0.5176-0.5296) |
| DBSLMM | h2f = 1.2 | 0.5326 (0.5266-0.5386) | 0.5236 (0.5176-0.5296) |

**Table S9.** Association of Different Polygenic Risk Scores (PRS) With Coronary Artery Disease (CAD) in Tuning Case-Control Study and in Prospective Cohort Study in Japan Biobank dataset

|  |  | **AUC (95% CI) PRS** | |
| --- | --- | --- | --- |
| **Methods** | **P-value/Tuning Parameters** | **Case-control study (N = 9,499)** | **Cohort Study (N = 7,036)** |
| Clumping and Thresholding | R2=0.02, P=0.00000005 | 0.549 (0.5454-0.5526) | 0.5341 (0.5312-0.5371) |
| Clumping and Thresholding | R2=0.02, P=0.000005 | 0.5529 (0.5494-0.5565) | 0.5395 (0.5365-0.5424) |
| Clumping and Thresholding | R2=0.02, P=0.0005 | 0.5602 (0.5567-0.5638) | 0.5488 (0.5458-0.5517) |
| Clumping and Thresholding | R2=0.02, P=0.05 | 0.5584 (0.5548-0.5619) | 0.542 (0.5391-0.545) |
| Clumping and Thresholding | R2=0.02, P=0.5 | 0.5559 (0.5523-0.5595) | 0.5401 (0.5372-0.5431) |
| Clumping and Thresholding | R2=0.02, P=1 | 0.5558 (0.5522-0.5594) | 0.5394 (0.5364-0.5423) |
| Clumping and Thresholding | R2=0.04, P=0.00000005 | 0.5492 (0.5456-0.5527) | 0.5331 (0.5301-0.536) |
| Clumping and Thresholding | R2=0.04, P=0.000005 | 0.5515 (0.5479-0.5551) | 0.5372 (0.5343-0.5402) |
| Clumping and Thresholding | R2=0.04, P=0.0005 | 0.558 (0.5545-0.5616) | 0.5472 (0.5443-0.5501) |
| Clumping and Thresholding | R2=0.04, P=0.05 | 0.567 (0.5634-0.5706) | 0.5489 (0.546-0.5519) |
| Clumping and Thresholding | R2=0.04. P=0.5 | 0.5647 (0.5611-0.5638) | 0.5448 (0.5418-0.5477) |
| Clumping and Thresholding | R2=0.04, P=1 | 0.5639 (0.5604-0.5675) | 0.5444 (0.5414-0.5477) |
| Clumping and Thresholding | R2=0.06, P=0.00000005 | 0.5471 (0.5435-0.5507) | 0.532 (0.5291-0.5349) |
| Clumping and Thresholding | R2=0.06, P=0.000005 | 0.5471 (0.5435-0.5507) | 0.532 (0.5291-0.5349) |
| Clumping and Thresholding | R2=0.06, P=0.0005 | 0.5522 (0.5486-0.5558) | 0.543 (0.54-0.5459) |
| Clumping and Thresholding | R2=0.06, P=0.05 | 0.5671 (0.5635-0.5707) | 0.5523 (0.5494-0.5553) |
| Clumping and Thresholding | R2=0.06, P=0.5 | 0.5683 (0.5647-0.5719) | 0.5488 (0.5458-0.5517) |
| Clumping and Thresholding | R2=0.06, P=1 | 0.5679 (0.5643-0.5715) | 0.548 (0.5451-0.551) |
| Clumping and Thresholding | R2=0.08, P=0.00000005 | 0.5495 (0.5459-0.5531) | 0.5339 (0.531-0.5369) |
| Clumping and Thresholding | R2=0.08, P=0.000005 | 0.5457 (0.5421-0.5493) | 0.5347 (0.5318-0.5377) |
| Clumping and Thresholding | R2=0.08, P=0.0005 | 0.551 (0.5474-0.5546) | 0.542 (0.539-0.5449) |
| Clumping and Thresholding | R2=0.08, P=0.05 | 0.567 (0.5634-0.5706) | 0.5551 (0.5522-0.5581) |
| Clumping and Thresholding | R2=0.08, P=0.5 | 0.5712 (0.5676-0.5748) | 0.552 (0.5491-0.5549) |
| Clumping and Thresholding | R2=0.08, P=1 | 0.5709 (0.5673-0.5745) | 0.5512 (0.5483-0.5542) |
| LDpred | p = 0.001 | 0.5313 (0.508-0.5543) | 0.5185 (0.4994-0.5376) |
| LDpred | p = 0.01 | 0.5327 (0.5096-0.5559) | 0.5181 (0.499-0.5372) |
| LDpred | p = 0.1 | 0.593 (0.5698-0.6161) | 0.569 (0.5499-0.5881) |
| **Methods** | **P-value/Tuning Parameters** | **Case-control study (N = 9,499)** | **Cohort Study (N = 7,036)** |
| LDpred | p = 1 | 0.582 (0.5589-0.6052) | 0.56 (0.5409-0.5791) |
| LDpred | p = 0.003 | 0.5311 (0.5079-0.5542) | 0.5192 (0.5001-0.5383) |
| LDpred | p = 0.03 | 0.5446 (0.5214-0.5677) | 0.5286 (0.5095-0.5477) |
| LDpred | p = 0.3 | 0.586 (0.5628-0.6091) | 0.5634 (0.5443-0.5825) |
| lassosum | s=0.2, lambda=0.001 | 0.5577 (0.5533-0.5622) | 0.5395 (0.5356-0.5434) |
| lassosum | s=0.2, lambda=0.0013 | 0.5626 (0.5582-0.5671) | 0.543 (0.5391-0.5469) |
| lassosum | s=0.2, lambda=0.0016 | 0.5688 (0.5644-0.5733) | 0.5472 (0.5433-0.5511) |
| lassosum | s=0.2, lambda=0.0021 | 0.5752 (0.5707-0.5796) | 0.5519 (0.548-0.5559) |
| lassosum | s=0.2, lambda=0.0026 | 0.5798 (0.5754-0.5843) | 0.5572 (0.5533-0.5611) |
| lassosum | s=0.2, lambda=0.0034 | 0.5837 (0.5793-0.5882) | 0.5631 (0.5592-0.567) |
| lassosum | s=0.2, lambda=0.0043 | 0.5873 (0.5829-0.5918) | 0.5685 (0.5646-0.5724) |
| lassosum | s=0.2, lambda=0.0055 | 0.5903 (0.5858-0.5947) | 0.5719 (0.5679-0.5758) |
| lassosum | s=0.2, lambda=0.007 | 0.5863 (0.5819-0.5908) | 0.5676 (0.5637-0.5715) |
| lassosum | s=0.2, lambda=0.0089 | 0.5796 (0.5752-0.5841) | 0.5596 (0.5557-0.5636) |
| lassosum | s=0.2, lambda=0.0113 | 0.5723 (0.5678-0.5767) | 0.5525 (0.5486-0.5564) |
| lassosum | s=0.2, lambda=0.0114 | 0.5656 (0.5612-0.5701) | 0.5464 (0.5424-0.5503) |
| lassosum | s=0.2, lambda=0.0183 | 0.5593 (0.5549-0.5638) | 0.5412 (0.5373-0.5451) |
| lassosum | s=0.2, lambda=0.0234 | 0.5549 (0.5505-0.5594) | 0.5387 (0.5348-0.5426) |
| lassosum | s=0.2, lambda=0.0298 | 0.5555 (0.551-0.56) | 0.54 (0.5361-0.5439) |
| lassosum | s=0.2, lambda=0.0379 | 0.5562 (0.5518-0.5607) | 0.54 (0.536-0.5439) |
| lassosum | s=0.2, lambda=0.0483 | 0.5311 (0.5266-0.5355) | 0.5191 (0.5152-0.523) |
| lassosum | s=0.2, lambda=0.0616 | 0.5311 (0.5266-0.5355) | 0.5191 (0.5152-0.523) |
| lassosum | s=0.2, lambda=0.0785 | 0.5311 (0.5266-0.5355) | 0.5191 (0.5152-0.523) |
| lassosum | s=0.2, lambda=0.1 | 0.5311 (0.5266-0.5355) | 0.5191 (0.5152-0.523) |
| lassosum | s=0.5, lambda=0.001 | 0.5684 (0.564-0.5729) | 0.5491 (0.5452-0.553) |
| lassosum | s=0.5, lambda=0.0013 | 0.5724 (0.568-0.5769) | 0.5519 (0.548-0.5558) |
| lassosum | s=0.5, lambda=0.0016 | 0.5772 (0.5728-0.5817) | 0.5552 (0.5513-0.5592) |
| lassosum | s=0.5, lambda=0.0021 | 0.5818 (0.5773-0.5862) | 0.5592 (0.5553-0.5631) |
| lassosum | s=0.5, lambda=0.0026 | 0.5853 (0.5809-0.5898) | 0.5636 (0.5597-0.5675) |
| lassosum | s=0.5, lambda=0.0034 | 0.5887 (0.5843-0.5932) | 0.569 (0.565-0.5729) |
| **Methods** | **P-value/Tuning Parameters** | **Case-control study (N = 9,499)** | **Cohort Study (N = 7,036)** |
| lassosum | s=0.5, lambda=0.0043 | 0.5921 (0.5876-0.5965) | 0.5736 (0.5697-0.5775) |
| lassosum | s=0.5, lambda=0.0055 | 0.594 (0.5895-0.5985) | 0.5749 (0.571-0.5788) |
| lassosum | s=0.5, lambda=0.007 | 0.5886 (0.5841-0.5931) | 0.5689 (0.565-0.5728) |
| lassosum | s=0.5, lambda=0.0089 | 0.5802 (0.5758-0.5847) | 0.5601 (0.5562-0.564) |
| lassosum | s=0.5, lambda=0.0113 | 0.5729 (0.5685-0.5774) | 0.5527 (0.5488-0.5566) |
| lassosum | s=0.5, lambda=0.0114 | 0.5663 (0.5618-0.5707) | 0.5469 (0.5429-0.5508) |
| lassosum | s=0.5, lambda=0.0183 | 0.5603 (0.5558-0.5647) | 0.5416 (0.5377-0.5455) |
| lassosum | s=0.5, lambda=0.0234 | 0.5556 (0.5512-0.5601) | 0.539 (0.5351-0.5429) |
| lassosum | s=0.5, lambda=0.0298 | 0.5566 (0.5521-0.561) | 0.5406 (0.5367-0.5446) |
| lassosum | s=0.5, lambda=0.0379 | 0.5563 (0.5518-0.5607) | 0.5399 (0.536-0.5438) |
| lassosum | s=0.5, lambda=0.0483 | 0.5311 (0.5266-0.5355) | 0.5191 (0.5152-0.523) |
| lassosum | s=0.5, lambda=0.0616 | 0.5311 (0.5266-0.5355) | 0.5191 (0.5152-0.523) |
| lassosum | s=0.5, lambda=0.0785 | 0.5311 (0.5266-0.5355) | 0.5191 (0.5152-0.523) |
| lassosum | s=0.5, lambda=0.1 | 0.5311 (0.5266-0.5355) | 0.5191 (0.5152-0.523) |
| lassosum | s=0.9, lambda=0.001 | 0.5806 (0.5761-0.585) | 0.5611 (0.5572-0.565) |
| lassosum | s=0.9, lambda=0.0013 | 0.5832 (0.5787-0.5876) | 0.5631 (0.5592-0.567) |
| lassosum | s=0.9, lambda=0.0016 | 0.5859 (0.5815-0.5904) | 0.5653 (0.5614-0.5692) |
| lassosum | s=0.9, lambda=0.0021 | 0.5884 (0.5839-0.5929) | 0.5682 (0.5642-0.5721) |
| lassosum | s=0.9, lambda=0.0026 | 0.591 (0.5866-0.5955) | 0.5716 (0.5677-0.5755) |
| lassosum | s=0.9, lambda=0.0034 | 0.5931 (0.5887-0.5976) | 0.5752 (0.5713-0.5792) |
| lassosum | s=0.9, lambda=0.0043 | 0.5944 (0.5899-0.5988) | 0.5777 (0.5738-0.5816) |
| lassosum | s=0.9, lambda=0.0055 | 0.5923 (0.5879-0.5968) | 0.5748 (0.5709-0.5787) |
| lassosum | s=0.9, lambda=0.007 | 0.5853 (0.5808-0.5898) | 0.5654 (0.5614-0.5693) |
| lassosum | s=0.9, lambda=0.0089 | 0.5771 (0.5727-0.5816) | 0.5568 (0.5528-0.5607) |
| lassosum | s=0.9, lambda=0.0113 | 0.5707 (0.5662-0.5751) | 0.5501 (0.5462-0.554) |
| lassosum | s=0.9, lambda=0.0114 | 0.5656 (0.5612-0.5701) | 0.546 (0.5421-0.5499) |
| lassosum | s=0.9, lambda=0.0183 | 0.559 (0.5545-0.5634) | 0.5405 (0.5366-0.5445) |
| lassosum | s=0.9, lambda=0.0234 | 0.5558 (0.5513-0.5602) | 0.5384 (0.5345-0.5423) |
| lassosum | s=0.9, lambda=0.0298 | 0.5578 (0.5533-0.5623) | 0.5405 (0.5366-0.5444) |
| lassosum | s=0.9, lambda=0.0379 | 0.5563 (0.5518-0.5608) | 0.5398 (0.5359-0.5438) |
| **Methods** | **P-value/Tuning Parameters** | **Case-control study (N = 9,499)** | **Cohort Study (N = 7,036)** |
| lassosum | s=0.9, lambda=0.0483 | 0.5311 (0.5266-0.5355) | 0.5191 (0.5152-0.523) |
| lassosum | s=0.9, lambda=0.0616 | 0.5311 (0.5266-0.5355) | 0.5191 (0.5152-0.523) |
| lassosum | s=0.9, lambda=0.0785 | 0.5311 (0.5266-0.5355) | 0.5191 (0.5152-0.523) |
| lassosum | s=0.9, lambda=0.1 | 0.5311 (0.5266-0.5355) | 0.5191 (0.5152-0.523) |
| lassosum | s=1, lambda=0.001 | 0.5723 (0.5678-0.5767) | 0.5572 (0.5533-0.5612) |
| lassosum | s=1, lambda=0.0013 | 0.5723 (0.5678-0.5768) | 0.5576 (0.5537-0.5615) |
| lassosum | s=1, lambda=0.0016 | 0.5721 (0.5676-0.5765) | 0.5579 (0.554-0.5618) |
| lassosum | s=1, lambda=0.0021 | 0.5713 (0.5669-0.5758) | 0.5578 (0.5539-0.5617) |
| lassosum | s=1, lambda=0.0026 | 0.5697 (0.5653-0.5742) | 0.5569 (0.5529-0.5608) |
| lassosum | s=1, lambda=0.0034 | 0.5672 (0.5628-0.5717) | 0.5548 (0.5508-0.5587) |
| lassosum | s=1, lambda=0.0043 | 0.5639 (0.5595-0.5684) | 0.5511 (0.5472-0.5551) |
| lassosum | s=1, lambda=0.0055 | 0.5599 (0.5554-0.5643) | 0.5464 (0.5425-0.5503) |
| lassosum | s=1, lambda=0.007 | 0.557 (0.5525-0.5614) | 0.5421 (0.5382-0.546) |
| lassosum | s=1, lambda=0.0089 | 0.5559 (0.5515-0.5604) | 0.5392 (0.5353-0.5431) |
| lassosum | s=1, lambda=0.0113 | 0.5574 (0.5529-0.5618) | 0.5379 (0.534-0.5418) |
| lassosum | s=1, lambda=0.0114 | 0.5577 (0.5532-0.5621) | 0.5373 (0.5333-0.5412) |
| lassosum | s=1, lambda=0.0183 | 0.5552 (0.5507-0.5596) | 0.5374 (0.5335-0.5413) |
| lassosum | s=1, lambda=0.0234 | 0.5513 (0.5469-0.5558) | 0.5343 (0.5304-0.5383) |
| lassosum | s=1, lambda=0.0298 | 0.5553 (0.5509-0.5598) | 0.5379 (0.534-0.5418) |
| lassosum | s=1, lambda=0.0379 | 0.5566 (0.5521-0.5611) | 0.54 (0.5361-0.544) |
| lassosum | s=1, lambda=0.0483 | 0.5311 (0.5266-0.5355) | 0.5191 (0.5152-0.523) |
| lassosum | s=1, lambda=0.0616 | 0.5311 (0.5266-0.5355) | 0.5191 (0.5152-0.523) |
| lassosum | s=1, lambda=0.0785 | 0.5311 (0.5266-0.5355) | 0.5191 (0.5152-0.523) |
| lassosum | s=1, lambda=0.1 | 0.5311 (0.5266-0.5355) | 0.5191 (0.5152-0.523) |
| PRS-CS | phi = 0.000001 | 0.583 (0.5619-0.604) | 0.5602 (0.5415-0.5788) |
| PRS-CS | phi =0.0001 | 0.597 (0.576-0.6181) | 0.5752 (0.5565-0.5939) |
| PRS-CS | phi = 0.01 | 0.5811 (0.5601-0.6022) | 0.5589 (0.5403-0.5776) |
| PRS-CS | phi = 1 | 0.5647 (0.5436-0.5858) | 0.5466 (0.5279-0.5652) |
| sBayesR | LD matrix = HapMap3 and 2.8M | 0.5308 (0.5248-0.5368) | 0.5186 (0.5126-0.5246) |
| sBayesR | LD matrix = 2.8M variants | 0.5328 (0.5268-0.5388) | 0.5177 (0.5117-0.5237) |
| **Methods** | **P-value/Tuning Parameters** | **Case-control study (N = 9,499)** | **Cohort Study (N = 7,036)** |
| LDpred-funct | Baseline-LD model with 75 annotations | 0.5907 (0.5847-0.5967) | 0.5692 (0.5632-0.5752) |
| DBSLMM | h2f = 0.8 | 0.5347 (0.5314-0.5381) | 0.5258 (0.5201-0.5314) |
| DBSLMM | h2f = 1 | 0.5353 (0.5319-0.5386) | 0.5263 (0.5207-0.532) |
| DBSLMM | h2f = 1.2 | 0.5358 (0.5324-0.5391) | 0.5268 (0.5212-0.5324) |

**Table S10.** C Statistics (Derived for Cox Regression) for CAD Using Recalibrated Models in the PCE Prospective Cohort, Primary Analysis. Results are shown for the European meta-analysis and Japan Biobank datasets. Results are presented for the full population and stratified by gender and age group (below or above 55 years of age). Results for the sensitivity analysis using only participants with no reported lipid-lowering treatment at baseline also shown.

| A. European Meta-Analysis | | | | | | |
| --- | --- | --- | --- | --- | --- | --- |
|  | **All Participants (N=272,307; 7,036 cases)** | **Men (N=124,155; 5093 cases)** | **Women (N=148,152; 1,943 cases)** | **< 55 years old (N=102,330; 1,276 cases)** | **≥ 55 years old (N=169,977; 5,760 cases)** | **Participants Not Receiving Lipid-Lowering Treatment at Baseline (N= 235,172; 5,091 cases)** |
| **PRS** | 0.636 (0.63-0.642) | 0.639 (0.632-0.647) | 0.638 (0.625-0.65) | 0.683 (0.669-0.698) | 0.629 (0.622-0.636) | 0.642 (0.634-0.649) |
| **PCE** | 0.718 (0.713-0.723) | 0.663 (0.656-0.67) | 0.706 (0.695-0.717) | 0.749 (0.736-0.761) | 0.665 (0.658-0.671) | 0.73 (0.724-0.737) |
| **PRS + PCE** | 0.752 (0.746-0.757) | 0.712 (0.705-0.718) | 0.74 (0.729-0.75) | 0.791 (0.779-0.803) | 0.704 (0.698-0.71) | 0.764 (0.758-0.77) |
| B. Japan Biobank Dataset | | | | | | |
|  | **All Participants (N=272,307; 7,036 cases)** | **Men (N=124,155; 5093 cases)** | **Women (N=148,152; 1,943 cases)** | **< 55 years old (N=102,330; 1,276 cases)** | **≥ 55 years old (N=169,977; 5,760 cases)** | **Participants Not Receiving Lipid-Lowering Treatment at Baseline (N= 235,172; 5,091 cases)** |
| **PRS** | 0.582 (0.576-0.589) | 0.582 (0.575-0.59) | 0.584 (0.572-0.597) | 0.625 (0.61-0.64) | 0.576 (0.568-0.583) | 0.583 (0.575-0.591) |
| **PCE** | 0.718 (0.713-0.723) | 0.663 (0.656-0.67) | 0.706 (0.695-0.717) | 0.749 (0.736-0.761) | 0.665 (0.658-0.671) | 0.73 (0.724-0.737) |
| **PRS + PCE** | 0.73 (0.725-0.735) | 0.681 (0.675-0.688) | 0.718 (0.707-0.729) | 0.767 (0.755-0.78) | 0.678 (0.671-0.684) | 0.743 (0.737-0.749) |

**Table S11.** C-statistics (Derived from Cox Regression) for CAD Using European Meta-analysis dataset and Recalibrated Models in the PCE Prospective Cohort, Primary Analysis. Results are presented for full population and stratified by gender and age group (below or above 55 years of age). Results for the sensitivity analysis using only participants with no reported lipid-lowering treatment at baseline also shown.

| A. Full Population (N=272,307; 7036 cases) | | | | | | | |
| --- | --- | --- | --- | --- | --- | --- | --- |
|  | **Clumping and Thresholding** | **LDpred** | **lassosum** | **PRS-CS** | **sBayesR** | **LDpred-funct** | **DBSLMM** |
| **PRS** | 0.603 (0.597-0.61) | 0.63 (0.624-0.636) | 0.628 (0.621-0.634) | 0.63 (0.624-0.636) | 0.603 (0.597-0.61) | 0.62 (0.614-0.627) | 0.523 (0.517-0.53) |
| **PCE** | 0.718 (0.713-0.723) | 0.718 (0.713-0.723) | 0.718 (0.713-0.723) | 0.718 (0.713-0.723) | 0.718 (0.713-0.723) | 0.718 (0.713-0.723) | 0.718 (0.713-0.723) |
| **PRS + PCE** | 0.738 (0.732-0.743) | 0.749 (0.743-0.754) | 0.748 (0.743-0.753) | 0.749 (0.744-0.754) | 0.738 (0.732-0.743) | 0.743 (0.738-0.749) | 0.718 (0.713-0.724) |
| B. Men (N=124,155; 5093 cases) | | | | | | | |
|  | **Clumping and Thresholding** | **LDpred** | **lassosum** | **PRS-CS** | **sBayesR** | **LDpred-funct** | **DBSLMM** |
| **PRS** | 0.606 (0.599-0.614) | 0.632 (0.624-0.639) | 0.63 (0.623-0.638) | 0.633 (0.625-0.64) | 0.606 (0.599-0.614) | 0.621 (0.613-0.628) | 0.515 (0.507-0.523) |
| **PCE** | 0.663 (0.656-0.67) | 0.663 (0.656-0.67) | 0.663 (0.656-0.67) | 0.663 (0.656-0.67) | 0.663 (0.656-0.67) | 0.663 (0.656-0.67) | 0.663 (0.656-0.67) |
| **PRS + PCE** | 0.688 (0.681-0.695) | 0.703 (0.697-0.71) | 0.703 (0.697-0.71) | 0.704 (0.697-0.711) | 0.688 (0.681-0.695) | 0.696 (0.69-0.703) | 0.661 (0.654-0.668) |
| C. Women (N=148,152; 1943 cases) | | | | | | | |
|  | **Clumping and Thresholding** | **LDpred** | **lassosum** | **PRS-CS** | **sBayesR** | **LDpred-funct** | **DBSLMM** |
| **PRS** | 0.602 (0.589-0.614) | 0.632 (0.619-0.644) | 0.622 (0.609-0.635) | 0.629 (0.617-0.642) | 0.602 (0.589-0.614) | 0.622 (0.61-0.635) | 0.527 (0.514-0.54) |
| **PCE** | 0.706 (0.695-0.717) | 0.706 (0.695-0.717) | 0.706 (0.695-0.717) | 0.706 (0.695-0.717) | 0.706 (0.695-0.717) | 0.706 (0.695-0.717) | 0.706 (0.695-0.717) |
| **PRS + PCE** | 0.725 (0.714-0.735) | 0.736 (0.726-0.747) | 0.732 (0.722-0.743) | 0.736 (0.726-0.747) | 0.725 (0.714-0.735) | 0.731 (0.72-0.742) | 0.705 (0.695-0.716) |
| D. Aged < 55 years old (N=102,330; 1276 cases) | | | | | | | |
|  | **Clumping and Thresholding** | **LDpred** | **lassosum** | **PRS-CS** | **sBayesR** | **LDpred-funct** | **DBSLMM** |
| **PRS** | 0.64 (0.625-0.656) | 0.675 (0.66-0.69) | 0.67 (0.655-0.685) | 0.675 (0.66-0.69) | 0.64 (0.625-0.656) | 0.662 (0.647-0.677) | 0.541 (0.525-0.557) |
| **PCE** | 0.749 (0.736-0.761) | 0.749 (0.736-0.761) | 0.749 (0.736-0.761) | 0.749 (0.736-0.761) | 0.749 (0.736-0.761) | 0.749 (0.736-0.761) | 0.749 (0.736-0.761) |
| **PRS + PCE** | 0.77 (0.757-0.782) | 0.783 (0.771-0.795) | 0.783 (0.77-0.795) | 0.784 (0.772-0.796) | 0.77 (0.757-0.782) | 0.779 (0.767-0.792) | 0.747 (0.734-0.76) |
| E. Aged ≥ 55 years old (N=169,977; 5,760 cases) | | | | | | | |
|  | **Clumping and Thresholding** | **LDpred** | **lassosum** | **PRS-CS** | **sBayesR** | **LDpred-funct** | **DBSLMM** |
| **PRS** | 0.599 (0.592-0.606) | 0.623 (0.616-0.63) | 0.621 (0.614-0.629) | 0.623 (0.616-0.63) | 0.599 (0.592-0.606) | 0.614 (0.607-0.621) | 0.522 (0.515-0.53) |
| **PCE** | 0.665 (0.658-0.671) | 0.665 (0.658-0.671) | 0.665 (0.658-0.671) | 0.665 (0.658-0.671) | 0.665 (0.658-0.671) | 0.665 (0.658-0.671) | 0.665 (0.658-0.671) |
| **PRS + PCE** | 0.685 (0.679-0.692) | 0.698 (0.692-0.704) | 0.697 (0.691-0.703) | 0.698 (0.692-0.705) | 0.685 (0.679-0.692) | 0.692 (0.685-0.698) | 0.664 (0.658-0.671) |
| F. Participants not receiving lipid-lowering treatment at baseline (N=235,172; 5,091 cases) | | | | | | | |
|  | **Clumping and Thresholding** | **LDpred** | **lassosum** | **PRS-CS** | **sBayesR** | **LDpred-funct** | **DBSLMM** |
| **PRS** | 0.598 (0.59-0.606) | 0.629 (0.621-0.636) | 0.627 (0.619-0.634) | 0.63 (0.622-0.637) | 0.517 (0.509-0.525) | 0.623 (0.616-0.631) | 0.521 (0.513-0.529) |
| **PCE** | 0.73 (0.724-0.737) | 0.73 (0.724-0.737) | 0.73 (0.724-0.737) | 0.73 (0.724-0.737) | 0.73 (0.724-0.737) | 0.73 (0.724-0.737) | 0.73 (0.724-0.737) |
| **PRS + PCE** | 0.746 (0.74-0.752) | 0.758 (0.752-0.764) | 0.757 (0.751-0.763) | 0.759 (0.753-0.765) | 0.73 (0.724-0.736) | 0.755 (0.749-0.761) | 0.73 (0.723-0.736) |

**Table S12.** C-statistics (Derived from Cox Regression) for CAD Using Japan Biobank dataset and Recalibrated Models in the PCE Prospective Cohort, Primary Analysis. Results are presented for full population and stratified by gender and age group (below or above 55 years of age). Results for the sensitivity analysis using only participants with no reported lipid-lowering treatment at baseline also shown.

| A. Full Population (N=272,307; 7036 cases) | | | | | | | |
| --- | --- | --- | --- | --- | --- | --- | --- |
|  | **Clumping and Thresholding** | **LDpred** | **lassosum** | **PRS-CS** | **sBayesR** | **LDpred-funct** | **DBSLMM** |
| **PRS** | 0.552 (0.545-0.558) | 0.568 (0.562-0.575) | 0.577 (0.57-0.583) | 0.574 (0.568-0.581) | 0.552 (0.545-0.558) | 0.569 (0.562-0.575) | 0.527 (0.52-0.533) |
| **PCE** | 0.718 (0.713-0723) | 0.718 (0.713-0723) | 0.718 (0.713-0723) | 0.718 (0.713-0723) | 0.718 (0.713-0723) | 0.718 (0.713-0723) | 0.718 (0.713-0723) |
| **PRS + PCE** | 0.722 (0.717-0.727) | 0.726 (0.72-0.731) | 0.727 (0.721-0.732) | 0.727 (0.722-0.733) | 0.722 (0.717-0.727) | 0.726 (0.721-0.732) | 0.718 (0.713-0724) |
| B. Men (N=124,155; 5093 cases) | | | | | | | |
|  | **Clumping and Thresholding** | **LDpred** | **lassosum** | **PRS-CS** | **sBayesR** | **LDpred-funct** | **DBSLMM** |
| **PRS** | 0.548 (0.54-0.556) | 0.566 (0.558-0.574) | 0.578 (0.571-0.586) | 0.573 (0.566-0.581) | 0.548 (0.54-0.556) | 0.565 (0.558-0.573) | 0.519 (0.511-0.527) |
| **PCE** | 0.663 (0.656-0.67) | 0.663 (0.656-0.67) | 0.663 (0.656-0.67) | 0.663 (0.656-0.67) | 0.663 (0.656-0.67) | 0.663 (0.656-0.67) | 0.663 (0.656-0.67) |
| **PRS + PCE** | 0.666 (0.659-0.673) | 0.671 (0.664-0.678) | 0.675 (0.668-0.682) | 0.674 (0.667-0.681) | 0.666 (0.659-0.673) | 0.671 (0.665-0.678) | 0.66 (0.653-0.667) |
| C. Women (N=148,152; 1943 cases) | | | | | | | |
|  | **Clumping and Thresholding** | **LDpred** | **lassosum** | **PRS-CS** | **sBayesR** | **LDpred-funct** | **DBSLMM** |
| **PRS** | 0.553 (0.54-0.566) | 0.571 (0.559-0.584) | 0.571 (0.559-0.584) | 0.575 (0.562-0.588) | 0.553 (0.54-0.566) | 0.574 (0.562-0.587) | 0.524 (0.511-0.538) |
| **PCE** | 0.706 (0.695-0.717) | 0.706 (0.695-0.717) | 0.706 (0.695-0.717) | 0.706 (0.695-0.717) | 0.706 (0.695-0.717) | 0.706 (0.695-0.717) | 0.706 (0.695-0.717) |
| **PRS + PCE** | 0.707 (0.697-0.718) | 0.713 (0.702-0.723) | 0.712 (0.701-0.723) | 0.714 (0.703-0.724) | 0.707 (0.697-0.718) | 0.714 (0.704-0.725) | 0.705 (0.694-0.716) |
| D. Aged < 55 years old (N=102,330; 1276 cases) | | | | | | | |
|  | **Clumping and Thresholding** | **LDpred** | **lassosum** | **PRS-CS** | **sBayesR** | **LDpred-funct** | **DBSLMM** |
| **PRS** | 0.586 (0.571-0.602) | 0.605 (0.589-0.62) | 0.618 (0.603-0.633) | 0.616 (0.601-0.632) | 0.586 (0.571-0.602) | 0.606 (0.591-0.622) | 0.55 (0.534-0.566) |
| **PCE** | 0.749 (0.736-0.761) | 0.749 (0.736-0.761) | 0.749 (0.736-0.761) | 0.749 (0.736-0.761) | 0.749 (0.736-0.761) | 0.749 (0.736-0.761) | 0.749 (0.736-0.761) |
| **PRS + PCE** | 0.755 (0.742-0.768) | 0.758 (0.745-0.77) | 0.762 (0.75-0.775) | 0.762 (0.75-0.775) | 0.755 (0.742-0.768) | 0.759 (0.747-0.772) | 0.748 (0.735-0.76) |
| E. Aged ≥ 55 years old (N=169,977; 5,760 cases) | | | | | | | |
|  | **Clumping and Thresholding** | **LDpred** | **lassosum** | **PRS-CS** | **sBayesR** | **LDpred-funct** | **DBSLMM** |
| **PRS** | 0.544 (0.537-0.552) | 0.562 (0.555-0.569) | 0.567 (0.56-0.574) | 0.567 (0.56-0.574) | 0.544 (0.537-0.552) | 0.562 (0.554-0.569) | 0.526 (0.518-0.533) |
| **PCE** | 0.665 (0.658-0.671) | 0.665 (0.658-0.671) | 0.665 (0.658-0.671) | 0.665 (0.658-0.671) | 0.665 (0.658-0.671) | 0.665 (0.658-0.671) | 0.665 (0.658-0.671) |
| **PRS + PCE** | 0.667 (0.66-0.674) | 0.671 (0.664-0.678) | 0.672 (0.666-0.679) | 0.673 (0.666-0.679) | 0.667 (0.66-0.674) | 0.672 (0.665-0.678) | 0.664 (0.658-0.671) |
| F. Participants not receiving lipid-lowering treatment at baseline (N=235,172; 5,091 cases) | | | | | | | |
|  | **Clumping and Thresholding** | **LDpred** | **lassosum** | **PRS-CS** | **sBayesR** | **LDpred-funct** | **DBSLMM** |
| **PRS** | 0.549 (0.542-0.557) | 0.566 (0.559-0.574) | 0.569 (0.562-0.577) | 0.572 (0.564-0.58) | 0.515 (0.507-0.523) | 0.566 (0.558-0.574) | 0.525 (0.517-0.533) |
| **PCE** | 0.73 (0.724-0.737) | 0.73 (0.724-0.737) | 0.73 (0.724-0.737) | 0.73 (0.724-0.737) | 0.73 (0.724-0.737) | 0.73 (0.724-0.737) | 0.73 (0.724-0.737) |
| **PRS + PCE** | 0.734 (0.727-0.74) | 0.738 (0.731-0.744) | 0.739 (0.733-0.745) | 0.739 (0.733-0.745) | 0.729 (0.723-0.735) | 0.738 (0.732-0.744) | 0.73 (0.724-0.736) |

**Table S13.** Risk Reclassification at 7.5% Threshold for CAD Using Recalibrated PCE and PCE + PRS Models Stratified by Gender and Age Group (below or above 55 years of age)

| **Men** | | | | | | | |
| --- | --- | --- | --- | --- | --- | --- | --- |
|  | |  | PCE + PRS | |  |  |  |
|  | |  | < 7.5% | ≥ 7.5% | % reclassified | Categorical NRI: | Continuous NRI: |
| PCE | < 7.5% | Cases | 2881 | 1129 | 22.3 | 0.1671 (0.1532 to 0.1762) | 0.238 (0.2131 to 0.2599) |
|  | ≥ 7.5% | Cases | 281 | 783 | 5.5 |  |  |
|  | < 7.5% | Noncases | 92944 | 10405 | 9.1 | -0.051 (-0.056 to -0.047) | 0.1722 (0.1634 to 0.1849) |
|  | ≥ 7.5% | Noncases | 4517 | 5894 | 4.0 |  |  |
| NRI in full population: | | | | | | 0.1161 (0.1046 to 0.124) | 0.4102 (0.377 to 0.4448) |
|  | IDI: | 0.0708 (0.0674 to 0.0743) | | |  |  |  |
| **Women** | | | | | | | |
|  | |  | PCE + PRS | |  |  |  |
|  | |  | < 7.5% | ≥ 7.5% | % reclassified | Categorical NRI: | Continuous NRI: |
| PCE | < 7.5% | Cases | 1803 | 88 | 4.6 | 0.0413 (0.0302 to 0.0497) | 0.0962 (0.0837 to 0.1349) |
|  | ≥ 7.5% | Cases | 10 | 30 | 0.5 |  |  |
|  | < 7.5% | Noncases | 140610 | 999 | 0.7 | -0.0056 (-0.0066 to -0.0045) | 0.3196 (0.3108 to 0.3611) |
|  | ≥ 7.5% | Noncases | 244 | 459 | 0.2 |  |  |
| NRI in full population: | | | | | | 0.0357 (0.0248 to 0.0431) | 0.4158 (0.4057 to 0.496) |
|  | IDI: | 0.0452 (0.0398 to 0.0507) | | |  |  |  |
| **Aged < 55 years old** | | | | | | | |
|  | |  | PCE + PRS | |  |  |  |
|  | |  | < 7.5% | ≥ 7.5% | % reclassified | Categorical NRI: | Continuous NRI: |
| PCE | < 7.5% | Cases | 1053 | 140 | 11.0 | 0.0889 (0.0701 to 0.0984) | 0.1683 (0.1063 to 0.2012) |
|  | ≥ 7.5% | Cases | 28 | 46 | 2.2 |  |  |
|  | < 7.5% | Noncases | 97435 | 1244 | 1.2 | -0.0076 (-0.0088 to -0.0066) | 0.3438 (0.2509 to 0.4) |
|  | ≥ 7.5% | Noncases | 497 | 401 | 0.5 |  |  |
| NRI in full population: | | | | | | 0.0813 (0.0626 to 0.0901) | 0.5121 (0.3776 to 0.6012) |
|  | IDI: | 0.0702 (0.0628 to 0.0776) | | |  |  |  |
| **Aged ≥ 55 years old** | | | | | | | |
|  | |  | PCE + PRS | |  |  |  |
|  | |  | < 7.5% | ≥ 7.5% | % reclassified | Categorical NRI: | Continuous NRI: |
| PCE | < 7.5% | Cases | 4093 | 902 | 15.7 | 0.123 (0.1192 to 0.1336) | 0.1897 (0.1698 to 0.2285) |
|  | ≥ 7.5% | Cases | 200 | 543 | 3.5 |  |  |
|  | < 7.5% | Noncases | 140310 | 8769 | 5.6 | -0.0359 (-0.0391 to -0.0344) | 0.1617 (0.1569 to 0.1827) |
|  | ≥ 7.5% | Noncases | 3163 | 4253 | 2.0 |  |  |
| NRI in full population: | | | | | | 0.087 (0.0843 to 0.0969) | 0.3514 (0.3304 to 0.4112) |
|  | IDI: | 0.0605 (0.0572 to 0.0639) | | |  |  |  |

**Table S14.** Descriptive Characteristics of Tuning (Case-Control) Set, Secondary Analysis AFR population (N=218)

|  | **Men** | **Women** |
| --- | --- | --- |
| **N** | 111 | 107 |
| **Age (years) (mean (SD)) *** | 55.3 (8.46) | 52.92 (8.17) |
| **Mean SBP (mmHg) (mean (SD)) *** | 135.09 (18.69) | 138.38 (19.36) |
| **Smoking category (%) *** |  |  |
| **Non-smoker** | 56 (50.5) | 81 (75.7) |
| **Ex-smoker** | 32 (28.8) | 13 (12.15) |
| **Current smoker** | 23 (20.7) | 13 (12.15) |
| **Type 1 diabetes (%) *** | 2 (1.8) | 5 (4.7) |
| **Type 2 diabetes (%) *** | 48 (43.2) | 38 (35.5) |
| **Blood pressure lowering medication (%) *** | 70 (63.1) | 54 (50.5) |
| **Cholesterol (mmol/L) (mean (SD)) *** | 4.29 (1.47) | 4.56 (1.25) |
| **HDL cholesterol (mmol/L) (mean (SD)) *** | 1.25 (0.28) | 1.5 (0.33) |
| **Lipid lowering medication (%) *** | 49 (44.1) | 36 (33.6) |

* Variables used in PCE. Current smokers vs non-current smokers and diabetes as a binary variable

**Table S15.** Descriptive Characteristics of PCE Prospective Cohort/Testing Set, Secondary Analysis AFR population (N= 6,753)

|  | **Full Population** | | **Incident CAD** | |
| --- | --- | --- | --- | --- |
|  | **Men** | **Women** | **Men** | **Women** |
| **N** | 2901 | 3852 | 46 | 42 |
| **Age (years) (Mean (SD))** | 51.26 (8.13) | 51.9 (7.93) | 55.61 (8.57) | 55.4 (8.41) |
| **Mean SBP (mmHg) (mean (SD))** | 136. 87 (18.56) | 138.12 (18.59) | 136.83 (16.83) | 135.08 (20.25) |
| **Smoking category (%)** |  |  |  |  |
| **Current** | 481 (16.6) | 356 (9.2) | 10 (21.7) | 6 (14.3) |
| **Non-current** | 2420 (83.4) | 3496 (90.8) | 36 (78.3) | 36 (85.7) |
| **Type 1 Diabetes (%)** | 57 (2.0) | 67 (1.7) | 7 (15.2) | 3 (7.1) |
| **Type 2 Diabetes (%)** | 606 (20.1) | 699 (18.1) | 21 (45.7) | 26 (62.9) |
| **Blood pressure lowering medication (%)** | 828 (28.5) | 1325 (34.4) | 23 (50) | 27 (64.3) |
| **Cholesterol (mmol/L) (mean (SD))** | 4.99 (1.26) | 5.17 (1.18) | 4.77 (1.39) | 4.95 (1.53) |
| **HDL cholesterol (mmol/L) (mean (SD))** | 1.3 (0.33) | 1.54 (0.36) | 1.32 (0.37) | 1.38 (0.38) |
| **Person-years of observation (mean (SD))** | 12.69 (1.8) | 12.73 (1.6) | 5.48 (2.21) | 5.22 (2.41) |
| **Lipid lowering medication (%)** | 395 (13.6) | 462 (12.0) | 13 (28.3) | 12 (28.6) |

**Table S16.** Descriptive Characteristics of the PCE Prospective Cohort and Excluded Participants, Secondary Analysis AFR population

|  | **Prospective cohort (N = 6753)** | | **Excluded (N = 1135) *** | | |
| --- | --- | --- | --- | --- | --- |
|  | Men | Women | Men | Women | Missing counts |
| **N** | 2901 | 3852 | 425 | 552 | 158 |
| **Age (years) (Mean (SD))** | 51.26 (8.13) | 51.9 (7.93) | 51.99 (8.49) | 51.56 (7.97) |  |
| **Mean SBP (mmHg) (mean (SD))** | 136. 87 (18.56) | 138.12 (18.59) | 137.14 (18.4) | 137.95 (18.96) | 23 |
| **Smoking category (%)** |  |  |  |  | 26 |
| **Current** | 481 (16.6) | 356 (9.2) | 68 (16.0) | 70 (12.7) |  |
| **Non-current** | 2420 (83.4) | 3496 (90.8) | 357 (84.0) | 482 (87.3) |  |
| **Type 1 Diabetes (%)** | 57 (2.0) | 67 (1.7) | 8 (1.9) | 10 (1.8) |  |
| **Type 2 Diabetes (%)** | 606 (20.1) | 699 (18.1) | 106 (24.9) | 104 (18.8) |  |
| **Blood pressure lowering medication (%)** | 828 (28.5) | 1325 (34.4) | 152 (35.8) | 177 (32.1) |  |
| **Cholesterol (mmol/L) (mean (SD))** | 4.99 (1.26) | 5.17 (1.18) | 4.9 (1.27) | 5.16 (1.15) | 504 |
| **HDL cholesterol (mmol/L) (mean (SD))** | 1.3 (0.33) | 1.54 (0.36) | 1.28 (0.3) | 1.54 (0.36) | 1058 |
| **Person-years of observation (mean (SD))** | 12.69 (1.8) | 12.73 (1.6) | 12.16 (3.4) | 12.69 (2.11) |  |
| **Lipid lowering medication (%)** | 395 (13.6) | 462 (12.0) | 68 (16.0) | 60 (10.9) |  |

* 158 participants excluded from calculations due to missing variables on gender

**Table S17.** C Statistics (Derived for Cox Regression) for CAD Using Recalibrated Models in the PCE Prospective Cohort, Secondary Analysis AFR Population. Results are shown for the European meta-analysis and Japan Biobank datasets. Results are presented for the full population and stratified by gender and age group (below or above 55 years of age). Results for the sensitivity analysis using only participants with no reported lipid-lowering treatment at baseline also shown.

| A. European Meta-Analysis | | | | | | |
| --- | --- | --- | --- | --- | --- | --- |
|  | **All Participants (N= 6,753; 88 cases)** | **Men (N= 2,901; 46 cases)** | **Women (N= 3,852; 42 cases)** | **< 55 years old (N=4,528; 42 cases)** | **≥ 55 years old (N=2,225; 46 cases)** | **Participants Not Receiving Lipid-Lowering Treatment at Baseline (N= 5,896; 63 cases)** |
| **PRS** | 0.545 (0.488-0.564) | 0.572 (0.49-0.653) | 0.6 (0.512-0.688) | 0.549 (0.461-0.637) | 0.551 (0.472-0.63) | 0.54 (0.471-0.609) |
| **PCE** | 0.714 (0.659-0.769) | 0.674 (0.595-0.753) | 0.734 (0.653-0.815) | 0.657 (0.572-0.742) | 0.721 (0.656-0.787) | 0.698 (0.628-0.768) |
| **PRS + PCE** | 0.715 (0.662–0.768) | 0.708 (0.636-0.78) | 0.734 (0.657-0.811) | 0.671 (0.587-0.755) | 0.7 (0.634-0.767) | 0.71 (0.643-0.777) |
| B. Japan Biobank Dataset | | | | | | |
|  | **All Participants (N= 6,753; 88 cases)** | **Men (N= 2,901; 46 cases)** | **Women (N= 3,852; 42 cases)** | **< 55 years old (N=4,528; 42 cases)** | **≥ 55 years old (N=2,225; 46 cases)** | **Participants Not Receiving Lipid-Lowering Treatment at Baseline (N= 5,896; 63 cases)** |
| **PRS** | 0.533 (0.472-0.595) | 0.497 (0.409-0.585) | 0.549 (0.463-0.634) | 0.551 (0.463-0.639) | 0.506 (0.417-0.595) | 0.522 (0.452-0.591) |
| **PCE** | 0.714 (0.659-0.769) | 0.674 (0.595-0.753) | 0.734 (0.653-0.815) | 0.657 (0.572-0.742) | 0.721 (0.656-0.787) | 0.698 (0.628-0.768) |
| **PRS + PCE** | 0.714 (0.66-0.769) | 0.668 (0.589-0.748) | 0.735 (0.653-0.816) | 0.666 (0.582-0.75) | 0.719 (0.653-0.785) | 0.697 (0.627-0.767) |

**Table S18.** Net Reclassification Improvement and Integrated Discrimination Improvement Results in Secondary Analysis of 6,753 African Ancestry Participants

|  | **No. of Participants** | **Continuous Net Reclassification Improvement** | | **Categorical Net Reclassification Improvement** | **Integrated Discrimination Improvement** |
| --- | --- | --- | --- | --- | --- |
| Cases | 88 | | 0.044 (0.036 to 0.052) | 0.0 (-.0.46 to 0.0) |  |
| Noncases | 6514 | | -0.046 (-0.054 to -0.038) | 0.0 (0.0 to 0.005) |  |
| Full Population | 6602 | | -0.002 (-0.203 to 0.198) | 0.0 (-0.041 to 0.001) | 0.0129 (-0.0076 to -0.0333) |
| Censored | 151 | |  |  |  |

**Table S19.** Descriptive Characteristics of Tuning (Case-Control) Set, Secondary Analysis EAS population (N=62)

|  | **Men** | **Women** |
| --- | --- | --- |
| **N** | 24 | 38 |
| **Age (years) (mean (SD)) *** | 56.21 (9.03) | 53.87 (7.42) |
| **Mean SBP (mmHg) (mean (SD)) *** | 138.73 (15.46) | 138.24 (19.13) |
| **Smoking category (%) *** |  |  |
| **Non-smoker** | 10 (41.7) | 31 (81.6) |
| **Ex-smoker** | 13 (54.2) | 6 (15.8) |
| **Current smoker** | 1 (4.1) | 1 (2.6) |
| **Type 1 diabetes (%) *** | 0 (0) | 0 (0) |
| **Type 2 diabetes (%) *** | 10 (41.7) | 10 (26.3) |
| **Blood pressure lowering medication (%) *** | 16 (66.7) | 11 (28.9) |
| **Cholesterol (mmol/L) (mean (SD)) *** | 3.94 (1.33) | 4.84 (1.25) |
| **HDL cholesterol (mmol/L) (mean (SD)) *** | 1.26 (0.24) | 1.42 (0.32) |
| **Lipid lowering medication (%) *** | 14 (58.3) | 12 (31.6) |

* Variables used in PCE. Current smokers vs non-current smokers and diabetes as a binary variable

**Table S20.** Descriptive Characteristics of PCE Prospective Cohort/Testing Set, Secondary Analysis EAS population (N= 2,212)

|  | **Full Population** | | **Incident CAD** | |
| --- | --- | --- | --- | --- |
|  | **Men** | **Women** | **Men** | **Women** |
| **N** | 744 | 1468 | 12 | 15 |
| **Age (years) (Mean (SD))** | 51.76 (8.1) | 52.38 (7.67) | 57.5 (8.75) | 62.73 (4.06) |
| **Mean SBP (mmHg) (mean (SD))** | 137.63 (19.52) | 138.66 (19.1) | 149.96 (19.82) | 131.97 (22.22) |
| **Smoking category (%)** |  |  |  |  |
| **Current** | 114 (15.3) | 81 (5.5) | 3 (25.0) | 1 (6.7) |
| **Non-current** | 630 (84.7) | 1387 (94.5) | 9 (75.0) | 14 (93.3) |
| **Type 1 Diabetes (%)** | 4 (0.5) | 9 (0.6) | 0 (0) | 0 (0) |
| **Type 2 Diabetes (%)** | 100 (13.4) | 134 (9.1) | 4 (33.3) | 5 (33.3) |
| **Blood pressure lowering medication (%)** | 147 (19.8) | 248 (16.9) | 6 (50.0) | 8 (53.3) |
| **Cholesterol (mmol/L) (mean (SD))** | 5.35 (1.19) | 5.56 (1.17) | 4.96 (1.61) | 5.54 (1.67) |
| **HDL cholesterol (mmol/L) (mean (SD))** | 1.25 (0.29) | 1.58 (0.37) | 1.21 (0.23) | 1.6 (0.36) |
| **Person-years of observation (mean (SD))** | 12.92 (1.79) | 12.98 (1.48) | 4.33 (2.6) | 5.02 (1.93) |
| **Lipid lowering medication (%)** | 79 (10.6) | 132 (9.0) | 4 (33.3) | 4 (26.7) |

**Table S21.** Descriptive Characteristics of the PCE Prospective Cohort and Excluded Participants, Secondary Analysis EAS population

|  | **Prospective cohort (N = 2212)** | | **Excluded (N = 364)** | | |
| --- | --- | --- | --- | --- | --- |
|  | Men | Women | Men | Women | Missing counts |
| **N** | 744 | 1468 | 121 | 243 |  |
| **Age (years) (Mean (SD))** | 51.76 (8.1) | 52.38 (7.67) | 51.4 (7.82) | 52.26 (7.64) |  |
| **Mean SBP (mmHg) (mean (SD))** | 137.63 (19.52) | 138.66 (19.1) | 136.13 (17.5) | 137.2 (17.93) | 2 |
| **Smoking category (%)** |  |  |  |  | 6 |
| **Current** | 114 (15.3) | 81 (5.5) | 19 (15.7) | 12 (4.9) |  |
| **Non-current** | 630 (84.7) | 1387 (94.5) | 99 (84.3) | 228 (95.1) |  |
| **Type 1 Diabetes (%)** | 4 (0.5) | 9 (0.6) | 1 (0.8) | 1 (0.4) |  |
| **Type 2 Diabetes (%)** | 100 (13.4) | 134 (9.1) | 24 (19.8) | 21 (8.7) |  |
| **Blood pressure lowering medication (%)** | 147 (19.8) | 248 (16.9) | 23 (19.0) | 42 (17.3) |  |
| **Cholesterol (mmol/L) (mean (SD))** | 5.35 (1.19) | 5.56 (1.17) | 4.0 (0.98) | 5.2 (0.7) | 130 |
| **HDL cholesterol (mmol/L) (mean (SD))** | 1.25 (0.29) | 1.58 (0.37) | 1.03 (0.23) | 1.27 (.21) | 354 |
| **Person-years of observation (mean (SD))** | 12.92 (1.79) | 12.98 (1.48) | 12.28 (3.41) | 12.93 (1.68) |  |
| **Lipid lowering medication (%)** | 79 (10.6) | 132 (9.0) | 16 (13.2) | 18 (7.4) |  |

**Table S22.** C-Statistics for Coronary Artery Disease for Full Population and Stratified by Sex and Age Group (Younger and Older than 55 Years of Age), Secondary Analysis EAS population

| **C-Statistic (95% CI)** | | | | | | |
| --- | --- | --- | --- | --- | --- | --- |
|  | **All** | **Men** | **Women** | **Participants Aged <55 y** | **Participants Aged ≥ 55 y** | **Participants Not Receiving Lipid-Lowering Treatment at Baseline** |
| **A. East Asian Ancestry** | | | | | | |
| Participants, No. | 2212 | 744 | 1468 | 1350 | 862 | 2001 |
| Cases, No. | 27 | 12 | 15 | 5 | 22 | 18 |
| Polygenic risk score | .672 (.58-.765) | .647 (.498-.795) | .685 (.592-.777) | .778 (.673-.884) | .662 (.557-.768) | .68 (.569-.791) |
| Pooled cohort equation | .774 (.706-.841) | .754 (.662-.846) | .782 (.699-.864) | .846 (.721-.971) | .633 (.518-.747) | .76 (.676-.845) |
| Polygenic risk score + pooled cohort equation | .799 (.726-.872) | .765 (.667-.864) | .815 (.728-.903) | .89 (.813-.967) | .699 (.586-.813) | .782 (.692-.873) |

**Table S23.** C Statistics (Derived for Cox Regression) for CAD Using Recalibrated Models in the PCE Prospective Cohort, Secondary Analysis EAS Population. Results are shown for the European meta-analysis and Japan Biobank datasets. Results are presented for the full population and stratified by gender and age group (below or above 55 years of age). Results for the sensitivity analysis using only participants with no reported lipid-lowering treatment at baseline also shown.

| A. European Meta-Analysis | | | | | | |
| --- | --- | --- | --- | --- | --- | --- |
|  | **All Participants (N= 2,212; 27 cases)** | **Men (N= 744; 12 cases)** | **Women (N= 1,468; 15 cases)** | **< 55 years old (N=1,350; 5 cases)** | **≥ 55 years old (N=862; 22 cases)** | **Participants Not Receiving Lipid-Lowering Treatment at Baseline (N= 2,001; 18 cases)** |
| **PRS** | .674 (.588-.76) | .665 (.529-.8) | .689 (.605-.773) | .822 (.756-.888) | .661 (.565-.758) | .677 (.574-.781) |
| **PCE** | .774 (.706-.841) | .754 (.662-.846) | .782 (.699-.864) | .846 (.721-.971) | .633 (.518-.747) | .76 (.676-.845) |
| **PRS + PCE** | .805 (.738-.871) | .778 (.687-.869) | .815 (.731-.9) | .905 (.846-.964) | .695 (.586-.804) | .789 (.707-.871) |
| B. Japan Biobank Dataset | | | | | | |
|  | **All Participants (N= 2,212; 27 cases)** | **Men (N= 744; 12 cases)** | **Women (N= 1,468; 15 cases)** | **< 55 years old (N=1,350; 5 cases)** | **≥ 55 years old (N=862; 22 cases)** | **Participants Not Receiving Lipid-Lowering Treatment at Baseline (N= 2,001; 18 cases)** |
| **PRS** | .615 (.519-.71) | .669 (.528-.81) | .592 (.479-.706) | .742 (.571-.913) | .62 (.52-.721) | .657 (.556-.758) |
| **PCE** | .774 (.706-.841) | .754 (.662-.846) | .782 (.699-.864) | .846 (.721-.971) | .633 (.518-.747) | .76 (.676-.845) |
| **PRS + PCE** | .773 (.706-.841) | .755 (.664-.846) | .781 (.699-.864) | .845 (.719-.971) | .633 (.518-.747) | .76 (.675-.845) |

**Table S24.** Net Reclassification Improvement and Integrated Discrimination Improvement Results in Secondary Analysis of 2,212 East Asian Ancestry Participants

|  | **No. of Participants** | **Continuous Net Reclassification Improvement** | | **Categorical Net Reclassification Improvement** | **Integrated Discrimination Improvement** |
| --- | --- | --- | --- | --- | --- |
| Cases | 27 | | 0.336 (-0.142 to 0.56) | 0.0385 (-0.12 to 0.032) |  |
| Noncases | 2151 | | 0.206 (-0.054 to 0.451) | -0.01 (-0.224 to 0.0) |  |
| Full Population | 2178 | | 0.542 (-0.0347 to 1.011) | 0.0285 (-0.12 to 0.019) | 0.0449 (-0.001 to 0.091) |
| Censored | 34 | |  |  |  |
