# Supplementary material for: Polygenic risk score improves the accuracy of a clinical risk score for coronary artery disease
Source: BMC Med. 2022 Nov 7;20:385. doi: 10.1186/s12916-022-02583-y (PMC9639312; doi:10.1186/s12916-022-02583-y)
Supplement: Supplementary file 5 — Additional file 5: Additional figures. Figure S1. Correlation matrix of PRS methods in tuning dataset, White British population. Figure S2. Calibration and recalibration plots in UK Biobank testing dataset. [file 12916_2022_2583_MOESM5_ESM.docx]

**Figure S1.** Correlation of PRSs Constructed by Different Methods in the Tuning Set of 9,499 Prevalent CAD cases and an Equal Number of Controls.

Abbreviations: PT and LDpredfun refer to the Clumping and Thresholding and LDpred-funct methods, respectively.

**
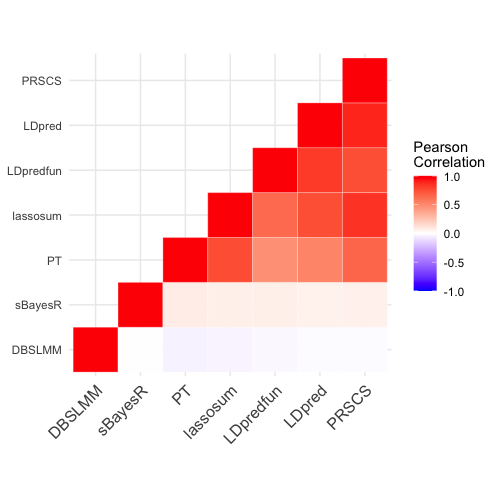
**

**Figure S2.** Calibration and Recalibration Plots Polygenic Risk Score for Coronary Artery Disease (CAD) and PRS Combined with PCE, Using a UK Biobank Prospective Cohort Sample. Model performance shown for non-recalibrated and recalibrated models. Recalibration was performed by fitting the predicted log hazard of the original models as a covariate in a Cox survival model. P_GND_ is the associated Greenwood-Nam-D’Agostino test P-value that tests a null hypothesis of the observed and expected probabilities
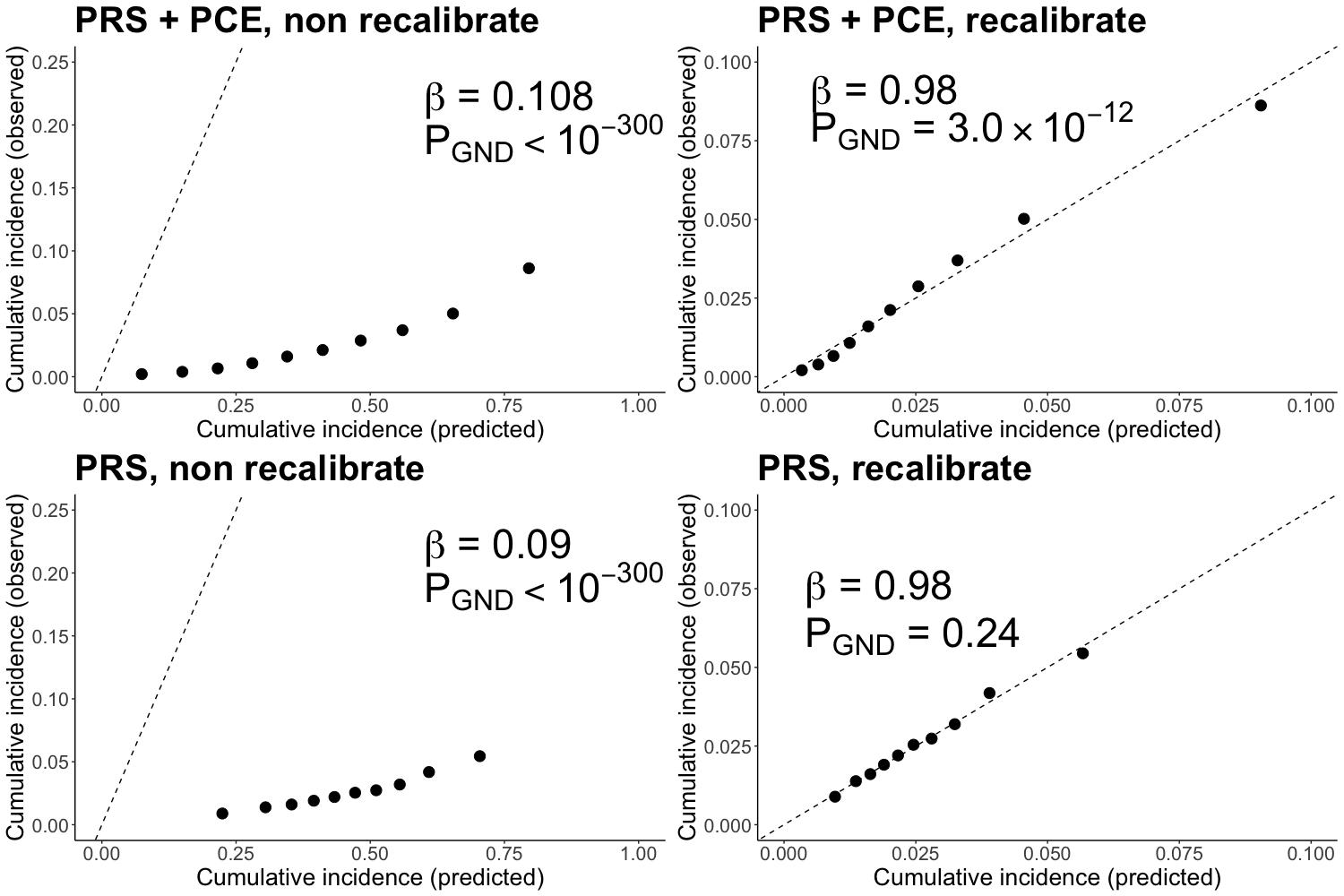
being identical in each group.
